# Older persons experiences of healthcare in rural Burkina Faso: Results of a cross sectional household survey

**DOI:** 10.1371/journal.pgph.0000193

**Published:** 2022-06-09

**Authors:** Ellen M. Goldberg, Mamadou Bountogo, Guy Harling, Till Baernighausen, Justine I. Davies, Lisa R. Hirschhorn

**Affiliations:** 1 University of Chicago Pritzker School of Medicine, Chicago, Illinois, United States of America; 2 Centre de Recherche en Santé de Nouna, Nouna, Burkina Faso; 3 Institute for Global Health, University College London, London, United Kingdom; 4 Heidelberg Institute of Global Health, University of Heidelberg, Heidelberg, Germany; 5 Institute of Applied Health Research, Birmingham University, Birmingham, United Kingdom; 6 Medical Social Sciences, Northwestern University Feinberg School of Medicine, Chicago, Illinois, United States of America; Instituto Nacional de Geriatria, MEXICO

## Abstract

Ensuring responsive healthcare which meets patient expectations and generates trust is important to increase rates of access and retention. This need is important for aging populations where non-communicable diseases (NCDs) are a growing cause of morbidity and mortality. We performed a cross-sectional household survey including socio-demographic; morbidities; and patient-reported health system utilization, responsiveness, and quality outcomes in individuals 40 and older in northwestern Burkina Faso. We describe results and use exploratory factor analysis to derive a contextually appropriate grouping of health system responsiveness (HSR) variables. We used linear or logistic regression to explore associations between socio-demographics, morbidities, and the grouped-variable, then between these variables and health system quality outcomes. Of 2,639 eligible respondents, 26.8% had least one NCD, 56.3% were frail or pre-frail and 23.9% had a recent healthcare visit, including only 1/3 of those with an NCD. Highest ratings of care experience (excellent/very good) included ease of following instructions (86.1%) and trust in provider skills (81.1%). The HSR grouping with the greatest factor loading included involvement in decision-making, clarity in communication, trust in the provider, and confidence in providers’ skills, labelled Shared Understanding and Decision Making (SUDM). In multivariable analysis, higher quality of life (OR 1.02,95%CI 1.01–1.04), frailty (OR 1.47,95%CI 1.00–2.16), and SUDM (OR 1.06,95%CI 1.05–1.09) were associated with greater health system trust and confidence. SUDM was associated with overall positive assessment of the healthcare system (OR 1.02,95%CI 1.01–1.03) and met healthcare needs (OR 1.09,95%CI 1.08–1.11). Younger age and highest wealth quintile were also associated with higher met needs. Recent healthcare access was low for people with existing NCDs, and SUDM was the most consistent factor associated with higher health system quality outcomes. Results highlight the need to increase continuity of care for aging populations with NCDs and explore strengthening SUDM to achieve this goal.

## Introduction

As access to care has improved in low and middle income country (LMIC) settings, understanding and ensuring the quality of this care has emerged as a critical step to reach effective universal health coverage and health-related sustainable development goals [1]. The Institute of Medicine (IOM) has defined six domains of quality, including effectiveness (often measured by technical quality), safety, timeliness, equity, efficiency, and patient-centeredness [2]. Patient-centeredness has been further emphasized through the World Health Organization’s (WHO) initiative for Integrated People Centered Health Care, which puts the patient at the center of the health care system, and is a core outcome in the framework from the Lancet Global Health Commission on High Quality Health Systems [1, 3].

Poor quality in any of the IOM domains is now a leading cause of preventable mortality, overtaking access as a major cause; poor quality contributes to a persistent equity gap and results in costs to the individual, health care system, and society [1, 4]. Gaps in quality are particularly apparent in non-communicable diseases (NCDs), which represent a growing burden across all countries as populations age [5]. Multiple studies are now showing the magnitude of gaps in both overall quality of care and resulting clinical outcomes (corresponding to technical outcomes, e.g. having a condition recognized and adequately managed) in NCDs and among older individuals [1, 6–10].

Receipt of person-centered care, a key IOM domain, and focus of initiatives to improve health care more broadly have been associated with improved healthcare utilization, better health outcomes, and patient safety. In contrast, poor experiences and perceived quality due to non-responsive care is associated with delay in accessing or returning to care or bypassing the formal care system, whether because of personal experience or through word-of-mouth [11–13]. Confidence and trust in the health system and overall satisfaction with care are also important quality outcomes of the care system, critical for ensuring willingness to access and return to care and consequently for the management of chronic conditions which are more frequent in populations as they age [14–16].

Measurement around patient-centeredness builds on the WHO Health Systems Responsiveness Framework which identified seven components of responsive outpatient care: dignity, confidentiality, involvement in decision making (autonomy), communication, choice of provider, prompt attention, and quality of basic amenities [17]. Larson directly linked health system responsiveness to experiential quality of care and proposed two areas for measurement: (1) patient experience of care, a process measure; and (2) patient satisfaction, a health system quality outcome measure of how well provided care meets patient needs and expectations [18]. The relationship between components of responsiveness of care and the health system quality outcomes is not well described, although recent work from Ghana found that higher reported responsiveness was associated with improved measures of outcomes including reported met medical needs (a measure of satisfaction with care) and confidence in the health care system [19].

As health burden and care needs continue to shift to individuals as they age and risk of NCDs increases, there is a need to expand the measurement of quality beyond providing technically correct treatment to care which is also empowering and meets these patients’ needs through shared decision making (SDM) [20]. SDM has been demonstrated to be important for improving self-management and care outcomes, including among people with risk factors for existing chronic conditions [21]. SDM involves the patient and provider collaborating through better communication to identify preferences and make treatment choices that meet the patient’s goals. This approach addresses health system responsiveness domains including autonomy, communication, and trust between the patient and provider.

Burkina Faso is one of the poorest countries in the world, with 43.8% of the population living in extreme poverty [22]. Health care expenditure as a percentage of GDP has increased since 2000, reaching 7% by 2016, but out of pocket sources still contribute a large amount to healthcare funding [23]. The population is aging and NCDs are increasing, now estimated to account for 24% deaths in Burkina Faso [24–30]. Gaps in both screening and care-seeking for NCDs and those at risk, including older individuals, is also noted to be of concern. For example, Cisse et al. reported rates of hypertension of close to 13% with very low rates of diagnosis and treatment and Wagner et al. reported low rates of care seeking among individuals with high cardiovascular risk factors [29, 30].

The formal public health system within the district level includes primary care centers (known as a Center for Health and Social Promotion (CSPS)) and a district hospital (known as a medical center with surgical antennae) as well as private clinics and pharmacies. Health services in Burkina Faso have historically been tailored towards maternal and child health and infectious diseases. However, there is increasing attention being given to NCDs, including establishment of an NCD division in Ministry of Health (MOH) and a national integrated NCD policy [31], as well as a strategic plan (2016–2020) which included goals of strengthening healthcare quality coordination for the elderly (M Bountogo, personal communication).

We describe the causes of recent healthcare seeking and reported experiences of care in public sector primary and secondary level facilities among adults aged 40 and older in Nouna, a rural region in Burkina Faso. These results are important for providers and policy makers in Burkina Faso and similar settings to facilitate improved experiences of care to increase care seeking and retention of the aging population and begin to reverse the growing burden of NCD-related morbidity and mortality. While there have been different definitions of the age cut-off for older adults, in Burkina Faso, life expectancy is 62 years, hence people 40 years of age or older are considered “older” in this study relative to the ages of other people in the population. This is also the age above which WHO recognizes people to be at increased risk of cardiovascular disease risk factors (for example diabetes and hypertension)–prominent NCDs of aging [32]. Addressing gaps in all domains of healthcare quality is required to respond to the needs of this aging population and reach the goals of global Healthy Aging agenda [33].

## Methods

### Study setting

The study was set in the Nouna Health and Demographic Surveillance System (HDSS) area, led by the Centre de Recherche en Santé de Nouna (CRSN) in the Boucle du Mouhoun region, north-western Burkina Faso. The demographic surveillance area of the Nouna HDSS consists of the market town of Nouna and 59 surrounding villages with a total population of 107 000 [34].

### Data collection

Data for this cross-sectional study were obtained during the baseline wave of the CRSN Heidelberg Aging Study (CHAS) and survey and data collection procedures have been described in detail elsewhere—the survey instrument is included as S1 Study Tool [28]. Briefly, we randomly sampled 4000 older adults (aged 40 years or older) from the 2015 CRSN census population. In villages with more than 90 adults aged 40 or older, a random sample of households with at least one age-eligible person was created, and one age-eligible adult in each selected household was randomly selected to complete the survey. In villages with fewer than 90 adults aged 40 or older, all households with one or more age-eligible individual were included. Data were collected using Open Data Kit (ODK) software on tablet computers at the participants’ houses between May and July 2018. Interviews were conducted either in French or translated into the local languages of Dioula or Mooré by the interviewers.

The household survey contained questions on sociodemographics; self-reported presence of diseases or other health conditions; visits to a healthcare provider for themselves in the last 3 months and facility-type last attended; reasons for last health facility visit; reasons for not attending a facility in the last three months; and selected measures of health system responsiveness and health system quality outcomes (Table 1). Other measures included Anxiety (measured using the Generalized Anxiety Disorder question (GAD-2) score) [35], depression (using Patient Health Questionnaire (PHQ- 9)) [36] and Quality of life (measured using the validated EuroHIS 8-item version of WHOQOL) [37]. Disability was measured using the 12 item WHO Disability Assessment Schedule, version 2 (WHODAS-II) disability score [38]. Both WHO DAS and QOL were scaled between 0–100 with 0 representing the lowest and 100 the highest values, as is standard for these scores [28]. Cognitive functioning was assessed using CSI-D [39]. The Fried frailty score was constructed as described previously [40].

**Table 1:** Sociodemographics, health conditions, reported medical care seeing and health system quality outcomes among individuals who attended versus did not attend a public primary or secondary level facility in the last visit 3 months prior to the survey.

		Overall population	Attended facility in last 3 months	Did not attend facility in last 3 months	P-value
	Group	N (%)	N (%)	N (%)	
Total		2639	632	2007	
Sex	Female	1315 (49.8)	338 (53.5)	977 (48.7)	0.035
Age	40–49	1141 (43.2)	271 (42.9)	870 (43.3)	0.079
	50–59	755 (28.6)	148 (23.4)	607 (30.2)	
	60–69	475 (18)	145 (22.9)	330 (16.4)	
	70–79	217 (8.2)	61 (9.7)	156 (7.8)	
	80+	53 (2)	11 (1.7)	42 (2.1)	
Educational attainment	No formal schooling	2215 (83.9)	515 (81.5)	1700 (84.7)	0.055
	Some education (any primary school or higher)	424 (16.1)	117 (18.5)	307 (15.3)	
Marital status	Widowed/divorced/single	606 (23)	164 (25.9)	442 (22)	0.041
	Married or cohabitating	2033 (77)	468 (74.1)	1565 (78)	
Wealth quintile	1 (least wealthy)	499 (18.9)	103 (16.3)	396 (19.7)	<0.0001*
	2	522 (19.8)	103 (16.3)	419 (20.9)	
	3	525 (19.9)	124 (19.6)	401 (20)	
	4	549 (20.8)	132 (20.9)	417 (20.8)	
	5 (most wealthy)	544 (20.6)	170 (26.9)	374 (18.6)	
Self-reported non-communicable diseases (NCD)†	Hypertension	463 (17.5)	171 (27.1)	292 (14.5)	
	Diabetes	62 (2.3)	18 (2.8)	44 (2.2)	
	Hypercholesterolemia	11 (.4)	7 (1.1)	4 (.2)	
	Heart disease	163 (6.2)	61 (9.7)	102 (5.1)	
	Stroke	36 (1.4)	12 (1.9)	24 (1.2)	
	Chronic respiratory disease	92 (3.5)	33 (5.2)	59 (2.9)	
	Cancer	14 (.5)	9 (1.4)	5 (.2)	
	≥1 NCD	708 (26.8)	250 (39.6)	458 (22.8)	<0.0001*
Self-reported TB or HIV†	TB	26 (1)	12 (1.9)	14 (.7)	
	HIV	16 (.61)	6 (.9)	10 (.5)	
	HIV and/or TB	41 (1.55)	17 (2.69)	24 (1.2)	0.0001*
Self-reported other conditions for > 3 months†	Cough	17 (.6)	8 (1.3)	9 (.4)	
	Headache or dizziness	50 (1.9)	16 (2.5)	34 (1.7)	
	Musculoskeletal or back pain	189 (7.2)	60 (9.5)	129 (6.4)	
	Dental	17 (.6)	5 (.8)	12 (.6)	
	Gastrointestinal	85 (3.2)	40 (6.3)	45 (2.2)	
	≥1 other condition	502 (19)	181 (28.6)	321 (16)	<0.0001*
Symptoms of mental health disorders (MHD)†	Cognitive impairment on testing	163 (6.2)	36 (5.7)	127 (6.3)	
	Symptoms of anxiety on testing	301 (11.4)	89 (14.1)	212 (10.6)	
	Depressive symptoms on testing	202 (7.7)	55 (8.7)	147 (7.3)	
	>1 MHD	518 (19.6)	142 (22.5)	376 (18.7)	0.039
Frailty	Not frail	1163 (44.1)	233 (36.9)	930 (46.3)	<0.0001*
	Frail/pre-frail	1476 (55.9)	399 (63.1)	1077 (53.7)	
Disability	WHO DAS s††	8.3 (0–20.8)	14.6 (4.2–27.1)	6.3 (0–18.8)	<0.0001*
Quality of life	WHO QoL ††	59.4 (46.9–65.6)	56.3 (43.8–65.6)	59.4 (46.9–68.8)	<0.0001*
Facility type for last visit	Center for Health and Social Promotion	2206 (83.6)	496 (78.5)	1710 (85.2)	<0.0001*
	District Hospital	433 (16.4)	136 (21.5)	297 (14.8)	
Financial access	Did not borrow or sell anything	2250 (85.3)	539 (85.3)	1711 (85.3)	0.98
	Borrowed or sold something to attend clinic	389 (14.7)	93 (14.7)	296 (14.7)	
Health System Quality Outcomes					
Reported met need**	Excellent	234 (8.9)	56 (8.9)	178 (8.9)	0.0058*
	Very Good	968 (36.7)	262 (41.5)	706 (35.2)	
	Good	1293 (49)	275 (43.5)	1018 (50.7)	
	Fair	116 (4.4)	33 (5.2)	83 (4.1)	
	Poor	28 (1.1)	6 (.9)	22 (1.1)	
Trust and confidence in health care system***	Very confident	872 (33)	246 (38.9)	626 (31.2)	0.0003*
	Somewhat confident	1610 (61)	358 (56.6)	1252 (62.4)	
	Not very confident	138 (5.2)	26 (4.1)	112 (5.6)	
	Not at all confident	19 (.7)	2 (.3)	17 (.8)	
Overall view of the health care system in this country****	Positive	1612 (61.1)	408 (64.6)	1204 (60)	
	Neutral	956 (36.2)	212 (33.5)	744 (37.1)	
	Poor	71 (2.7)	12 (1.9)	59 (2.9)	0.040

† P value represents ≥1 condition versus none or patients with HIV and/or TB versus none using chi square.

†† Scale from 0–100, median (IQR).

* P < 0.05 when adjusted for multiple comparisons using a Bonferroni correction.

** comparing excellent and very good versus others

*** Very confident versus others

****Positive versus others.

Table created by authors from the CRSN Heidelberg Aging Study.

### Definition of variables

#### Health system responsiveness and health system quality outcomes

A subset of all possible health system responsiveness domains was included due to constraints of the survey length. Questions were selected based on discussion between investigators and their perceived relevance to the local context and focus on experiential quality. They were taken from published studies in sub-Saharan Africa (Table A in S1 Appendix) [19, 41, 42]. Health system quality outcome questions included trust and confidence in receiving effective treatment, patient satisfaction (how well the received care met health need), and the overall view of the health system.

#### Demographic characteristics

Marital status was categorized as married/cohabiting versus single/widowed/divorced. Educational level was dichotomized as no education or any education (any primary school or higher). Participants were asked 37 questions on household assets and dwelling characteristics; from these, wealth quintiles were derived from the Filmer and Pritchett first principal component method [43]. Age was categorized in 10-year groups for the descriptive and univariate analysis and as a continuous variable in the multivariable analyses.

#### Disease categories

We included several self-reported conditions including non-communicable conditions (hypertension, diabetes, hypercholesterolemia, heart disease, stroke, chronic respiratory disease, and epilepsy), and communicable diseases (HIV and tuberculosis (TB)). Self-reported chronic symptoms (lasting for more than 3 months) included cough, headache, musculoskeletal or back pain, dental, or gastrointestinal manifestations. Some health conditions were captured as free text; these were translated and categorized through discussions among authors where necessary.

Participants were defined as having symptoms of anxiety based on a GAD-2 score ≥3, depression based on PHQ-9 score ≥ 10, and cognitive functioning was defined as possible/probable cognitive impairment for CSI-D score <7. Participants with at least one symptom of anxiety, depression, or cognitive impairment on testing were categorized as having a neurological or mental health diagnosis. WHODAS-II and quality of life were normalized to 0–100. For frailty, participants were dichotomized as robust versus prefrail/frail/unable to complete assessment.

## Analysis

### Analytic sample

We limited our sample to those who sought care at their last visit from a public sector primary (Center for Health and Social Promotion) or secondary level (District Hospital) facility to reflect our focus on local care seeking and the most common sources of care (93% of individuals for the variables of interest (see CONSORT diagram S1 Fig)). Using unweighted data, we described demographic characteristics, disease state, visit characteristics, and health system outcomes both among the whole sample surveyed and separately for participants who recently sought care (within the last 3 months) and those who did not. We used a Bonferroni correction to adjust for multiple comparisons.

### Health system responsiveness and health system quality outcomes among recent care users

We conducted an exploratory factor analysis of the experiential quality questions (S2 Fig) to explore grouping of these variables based on our assumption that one or more common constructs related to engagement in care and health system quality outcomes underlay our observed variables. We first scaled all HSR variables from 0 to 1, 1 representing the highest possible rating with wait time capped at 4 hours and consultation time capped at 1 hour based on a histogram of responses. We then ran a factor analysis with the scaled HSR variables as a measure of construct validity and used an eigenvalue cutoff of ≥1.0 for retained factors. We used a factor loading cutoff of ≥0.40 for individual variables within the qualifying factors. We used the resultant composite variable in subsequent analyses by scaling each individual variable to 0–100 with 0 representing the lowest and 100 the highest possible rating and averaged them to arrive at a final variable between 0–100.

### Bivariate analyses

We described individual HSR process ratings among recent care seekers. We limited these analyses to individuals with a visit in the last 3 months to reduce recall bias as we were not able to determine if less recent visits had occurred more than a year ago, which is the maximum duration used when assessing HSR [17]. We then tested for bivariate associations between demographic characteristics, health status (one or more self-reported NCD, one or more self-reported “other” condition, one or more symptom of mental health disorder, quality of life, frailty, and disability), facility type, financial access, wait time, and the HSR-group variable. We conducted similar analyses including the HSR-group variable with each of the three health system quality outcomes as the dependent variable of interest. Finally, we compared HSR variables dependent on whether the health facility visit occurred more or less than three months ago.

### Multivariable analyses

We ran logistic regressions and a generalized linear model regression (with a gaussian distribution and log link) for health system quality outcomes and the HSR-group variable respectively. Variables that met an inclusion criterion of P < 0.2 in the bivariate analyses were included. We also included age, sex, educational attainment, and wealth quintile, given their associations with reported experiential quality and selected health systems quality outcomes in previous studies [19, 44–46].

All statistical analyses were performed using Stata software (version 15.1; StataCorp LLC, College Station, Texas).

IRB: Ethical approval was obtained from Ethics Commission of the medical faculty Heidelberg (S-120/2018), the Burkina Faso Comité d’Ethique pour la Recherche en Santé (CERS) in Ouagadougou (2018-4-045) and the Institutional Ethics Committee (CIE) of the CRSN (2018–04). CRSN colleagues approached village leadership identified through existing channels (e.g., from the census and past studies), informed them about the study aims and activities, and obtained approval to come into the village to conduct the work. Written informed consent was obtained from each participant and a literate witness assisted in cases of illiteracy. Participants with abnormal results were contacted and referred for clinical care based on specifications determined in collaboration with the health system authorities. Medical services were also alerted of the conduct of the study and that they may receive patients as a result of the study.

Patients or the public were not involved in the design, conduct, reporting, or dissemination plans of our research.

## Results

### Population

Overall, 3,028 individuals responded to the survey including questions about care seeking with 177 excluded for missing data and 212 for care at a private sector facility or tertiary care hospital (S1 Fig). Among the 2,639 who reported their last visit to a public sector primary or secondary level facility, 632 (23.9%) sought care at one of these facilities in the 3 months prior to the survey (Table 1). Overall, one half (50%) were women, with 42.8% age 40–49 and 10.5% age 70 or older. Education was low (83.8% reported no formal education), and three quarters (76.4%) were married or cohabitating. One quarter reported at least one NCD (26.8%), with lower rates of communicable diseases such as HIV or TB (2.8%). The median WHO DAS score was 8.3 (interquartile range (IQR) 0–22.9) and for QoL was 59.4 (IQR 46.9–65.6), while 56.3% were categorized as frail or pre-frail.

Individuals who attended care in the last 3 months were significantly wealthier than those who did not attend care in this timeframe, and were more likely to have at least one NCD, either HIV or TB or both, or other conditions lasting for more than 3 months. Despite individuals with chronic diseases having attended clinic more recently, 65% of respondents with these conditions did not report attending care in this timeframe, including 62.7% of patients reporting hypertension and 66.7% of individuals reporting diabetes.

People who had attended in the last 3 months also had significantly higher disability measured by DAS scores (14.6 versus 6.3), lower QoL (56.3 versus 59.4), and were more likely to be frail or pre-frail (63.1% versus 53.7%) than those with no visits in the last 3 months; all p < .0001.

### Visits characteristics

The most common reasons overall for seeking care were for acute conditions (79.1%) including fever or malaria (51.6%), musculoskeletal pain (9.6%), and diarrhea or stomach-ache (8.4%). Chronic conditions accounted for care seeking in 12.9% including hypertension (6.2%), other cardiac conditions (2.1%) and diabetes (0.6%) (Table B in S1 Appendix). The most common reasons for care-seeking within the past 3 months were fever or malaria (37.8), high blood pressure (12.8%), musculoskeletal pain (12.0%), complaints related to the ear, nose, or throat (7.4%), or diarrhea or stomach-ache (7.0%). Not being sick was the most frequent reason for no recent care-seeking (87.3%) (Table C in S1 Appendix). Among those who stated other reasons for not seeking care, cost was the most common reason (50.4%), followed by preferring to see a traditional healer (11.6%) and poor previous experiences with the health system (6.0%).

### Health system quality outcomes

Overall, 32.7% of respondents were very confident that if they got sick, the health system could meet their needs. Compared with individuals with a visit over 3 months ago, individuals with recent visits had higher trust and confidence in the health system to provide effective care if they were sick (38.3% versus 30.8% very confident, p < .0004), although rates remained low. No differences were seen in their needs being met from their last visit or in overall opinion of the national health system (Table 1).

### Experiences of care at facilities (Health system responsiveness variables)

Among individuals with a visit to a public sector primary (CSPS) or secondary level (district hospital) public facility in the last 3 months, the median wait time was 20 minutes (IQR 10–30) while time spent with the provider was 15 minutes (IQR 10–25). Financial access was a challenge with 14.7% borrowing money or selling something to pay for health care. The highest ratings of experience of care (defined as excellent or very good) were in ease of following instructions (86.1%) and trust in the skills and abilities of the facility providers (81.1%). Lower ratings were seen for provider medical knowledge and skills (51.2%), clarity of communications (48.2%), with the lowest ratings in involvement in decision making (30.7%) (Table 2). Individuals without a recent facility visit reported lower ratings in clarity of communication, involvement in decision making, and trust in the skills and ability of the providers from their last visit (see S1 Appendix).

**Table 2:** Experience at last visit to a public sector primary or secondary level facility in the 3 months prior to survey.

Measure	Rating	N (%)
Clarity of provider communication	Excellent	45 (7.1)
	Very Good	260 (41.1)
	Good	287 (45.4)
	Fair	39 (6.2)
	Poor	1 (.2)
Ease of following provider advice	Excellent	81 (12.8)
	Very Good	463 (73.3)
	Good	76 (12)
	Fair	11 (1.7)
	Poor	1 (.2)
Provider medical knowledge and skills	Excellent	64 (10.1)
	Very Good	259 (41)
	Good	280 (44.3)
	Fair	27 (4.3)
	Poor	2 (.3)
Trust in skills and abilities of health workers at the facility	Very much	104 (16.5)
	Quite a bit	408 (64.6)
	Some	105 (16.6)
	Very little	13 (2.1)
	Not at all	2 (.3)
Involvement in decision making	Excellent	35 (5.5)
	Very Good	159 (25.2)
	Good	268 (42.4)
	Fair	101 (16)
	Poor	69 (10.9)
Shared Understanding and Decision Making (SUDM)	Median (Interquartile range)	62.5 (50–75)
Borrowed money or sold anything to pay for health care	Yes	93 (14.7)
	
	No	539 (85.3)
Wait time (median, IQR)		20 (10–30)

Table created by authors from the CRSN Heidelberg Aging Study.

The variable grouping with the greatest factor loading (the HSR-group variable) combined the results for questions on involvement in decision-making (autonomy), clarity in communication, trust in the provider, and confidence in providers’ skills (factor loadings of 0.44, 0.73, 0.57, and 0.69, respectively) (S2 Fig). After discussion between authors, we agreed that these variables reflected components necessary for shared understanding and decision making and termed the resultant variable as such (SUDM). We used the scaled variable as described in the methods and chose to not weight variable components as all were assumed to be equally important for SUDM. The median score for SUDM was 58.3 (Interquartile range (IQR) 50–75). In a multivariable analysis, only being seen in a district hospital was associated with higher SUDM (regression coefficient (β) 5.91 (95% CI 2.87–8.96)) (Table 3).

**Table 3:** Bivariate and multivariable analysis of factors associated with higher shared understanding and decision making (SUDM) among individuals with a visit to a primary or secondary level public sector facility in the 3 months prior to the survey.

		Bivariate analysis coefficient (95% CI)	P value	Multivariable analysis Coefficient (95% CI)	P value
Sex	Male	Reference		Reference	
	Female	-1.74 (-4.01–0.54)	0.13	-2.43 (-4.76 - -0.11)	0.040
Age (per year)		0.12 (0.016–0.22)	0.023	0.072 (-0.046–0.19)	0.23
Educational attainment	No formal schooling	Reference		Reference	
	Some education	0.32 (-2.61–3.24)	0.83	-0.57 (-3.64–2.50)	0.72
Marital status	Widowed/divorced/single	Reference			
	Married/cohabitating	-0.44 (-3.03–2.15)	0.74		
Wealth quintile†	1	Reference		Reference	
	2	0.24 (-3.87–4.36)	0.91	-0.33 (-4.28–3.62)	0.87
	3	0.37 (-3.45–4.19)	0.85	0.15 (-3.64–3.94)	0.94
	4	-0.76 (-4.64–3.12)	0.70	-1.12 (-4.88–2.64)	0.56
	5	1.38 (-2.31–5.07)	0.46	-0.13 (-3.87–3.60)	0.95
Facility type	Center for Health and Social Promotion	Reference		Reference	
	**Medical Center with Surgical Antenna**	**6.04 (3.32–8.76)**	**<0.001**	**5.48 (2.58–8.38)**	**< 0.001**
Financial Accessibility	Borrowed or sold anything to attend clinic	Reference			
	Did not borrow or sell anything	1.06 (-2.14–4.27)	0.56		
Non-communicable diseases (NCD)	No NCDs	Reference		Reference	
	≥1 NCD	1.94 (-0.38–4.26)	0.10	1.28 (-1.09–3.64)	0.29
TB or HIV	No TB or HIV	Reference			
	TB and/or HIV	-4.05 (-11.07–2.96)	0.26		
Other conditions for > 3 months	No other conditions	Reference			
	≥1 other condition	-0.88 (-3.39–1.63)	0.49		
Mental health disorders (MHD)	No MHDs	Reference		Reference	
	≥1 MHD	2.51 (-0.20–5.23)	0.070	1.53 (-1.39–4.45)	0.30
Frailty	Not frail	Reference			
	Pre-frail/Frail	1.16 (-1.19–3.51)	0.33		
Disability	WHO DAS score††	0.063 (0.00077–0.12)	0.047	0.013 (-0.06–0.09)	0.74
Quality of life	WHO QoL score ††	-0.036 (-0.11–0.039)	0.34		

Table created by authors from the CRSN Heidelberg Aging Study.

### Factors associated with health system quality outcomes

In the multivariable analysis, higher quality of life (OR 1.02, 95% CI 1.01–1.04), frailty (OR 1.47, 95% CI 1.00–2.16), and SUDM (OR 1.06, 95% CI 1.05–1.09) were all associated with greater trust and confidence in the health system (Table 4). SUDM was associated with overall positive assessment of the health care system in Burkina Faso (OR 1.02, 95% CI 1.01–1.03) and met healthcare needs in the last visit (OR 1.09, 95% CI 1.08–1.11). Younger age and highest wealth quintile were also associated with higher scores for met needs, while having at least one mental health condition was associated with less positive ratings of the overall health system.

**Table 4:** Multivariable regression for health system quality outcomes among respondents with a visit to a public sector primary or secondary level facility in the 3 months prior to the survey.

		Trust and Confidence in healthcare system		Overall view of healthcare system		Health care needs met	
		Bivariate analysis	Multivariable analysis	Bivariate analysis	Multivariable analysis	Bivariate analysis	Multivariable analysis
		OR (95% CI)	OR (96% CI)	OR (95% CI)		OR (95% CI)	
Sex	Male	Reference	Reference	Reference	Reference	Reference	Reference
	Female	0.73 (0.53–1.01)	0.83 (0.58–1.19)	0.89 (0.64–1.23)	0.99 (0.70–1.40)	0.86 (0.63–1.17)	1.03 (0.71–1.49)
Age*		1.00 (0.98–1.01)	1.00 (0.98–1.01)	1.00 (0.99–1.01)	1.01 (0.99–1.03)	0.99 (0.98–1.01)	**0.98 (0.97–1.00)**
Education	No formal schooling	Reference	Reference	Reference	Reference	Reference	Reference
	Some education	1.02 (0.68–1.54)	0.97 (0.60–1.56)	0.93 (0.61–1.42)	1.07 (0.67–1.70)	1.09 (0.73–1.63)	0.92 (0.56–1.50)
Wealth quintile	1	Reference	Reference	Reference	Reference	Reference	Reference
	2	1.58 (0.90–2.79)	1.49 (0.79–2.81)	1.00 (0.56–1.77)	1.00 (0.55–1.80)	1.17 (0.68–2.03)	1.24 (0.65–2.35)
	3	1.86 (1.08–3.21)	1.60 (0.87–2.94)	1.05 (0.61–1.82)	1.03 (0.58–1.82)	1.53 (0.90–2.58)	1.58 (0.85–2.94)
	4	1.13 (0.66–1.96)	0.99 (0.53–1.83)	1.24 (0.71–2.14)	1.27 (0.72–2.25)	1.19 (0.71–2.00)	1.25 (0.68–2.30)
	5	1.31 (0.78–2.20)	0.88 (0.48–1.63)	0.77 (0.46–1.27)	0.77 (0.44–1.34)	1.83 (1.11–2.99)	**1.85 (1.00–3.42)**
Facility	CSPS	Reference		Reference	Reference	Reference	Reference
	District Hospital	1.13 (0.77–1.66)		0.68 (0.46–1.00)	0.70 (0.46–1.08)	1.43 (0.98–2.10)	0.90 (0.56–1.45)
Financial access	Did not borrow/sell anything	Reference		Reference		Reference	
	Borrowed/sold something	0.94 (0.60–1.48)		0.85 (0.54–1.33)		1.24 (0.80–1.92)	
NCDs	None	Reference		Reference		Reference	
	≥1 NCD	1.05 (0.76–1.45)		0.86 (0.61–1.19)		1.21 (0.88–1.67)	
HIV or TB	None	Reference	Reference	Reference		Reference	
	HIV and/or TB	0.47 (0.15–1.47)	0.64 (0.19–2.18)	0.77 (0.29–2.08)		0.53 (0.19–1.45)	
MHD	None	Reference		Reference	**Reference**	Reference	
	≥1 MHD	1.07 (0.73–1.56)		0.51 (0.35–0.74)	**0.52 (0.34–0.80)**	1.18 (0.81–1.72)	
Frailty	Not frail	Reference	**Reference**	Reference	Reference	Reference	
	Pre-frail/Frail	1.32 (0.95–1.85)	**1.47 (1.00–2.16)**	0.75 (0.53–1.06)	0.83 (0.57–1.21)	1.12 (0.81–1.55)	
Disability (DAS)	DAS score††	1.00 (0.99–1.01)		0.99 (0.98–1.00)	0.99 (0.98–1.01)	1.00 (0.99–1.01)	
QOL	QoL score ††	1.02 (1.01–1.03)	**1.02 (1.01–1.04)**	1.00 (0.99–1.01)		1.01 (0.99–1.02)	
Wait time		0.64 (0.18–2.27)		2.18 (0.61–7.78)		1.19 (0.34–4.12)	
SUDM		1.06 (1.05–1.07)	**1.06 (1.05–1.09)**	1.02 (1.00–1.03)	**1.02 (1.01–1.03)**	1.09 (1.07–1.11)	**1.09 (1.08–1.11)**

*per year.

CSPS: Center for Health and Social Promotion, NCD: Non communicable diseases, MHD: Mental health Disorder, QOL: Quality of Life SUDM: Shared Understanding and Decision making.

Table created by authors from the CRSN Heidelberg Aging Study.

## Discussion

Ensuring longitudinal preventive, promotive, and curative primary care among older adults in resource constrained settings is critical to reducing the burden of morbidity and mortality related to NCDs. In this household survey of individuals aged 40 or older in Nouna, Burkina Faso, we found that while about one-quarter of individuals sought care at a public primary or secondary care facility in the last three months, significant gaps existed in care seeking among individuals with NCDs or frailty. In addition to healthcare needs and wealth, we also found that higher ratings of health system quality outcomes were associated with care seeking behavior.

Acute conditions were the most common reason for care seeking among this older population overall, with just one-fifth of recent care seeking for more chronic conditions. However, care seeking overall was low—only one third of patients who self-reported an NCD and 41% of those with TB or HIV had a visit in the last three months, despite recommendations from many institutions including the World Health Organization that individuals with NCDs be seen at least every three months [32]. The lack of recent visits for individuals with chronic conditions requiring longitudinal care is of concern given the importance of ongoing management even when symptoms are not present. A study of the hypertension care cascade in Burkina Faso found that only 17.5% of patients with elevated blood pressure were aware of their diagnosis, and less than half were on treatment [29]. These gaps in both awareness and care are similar to other countries in the region including Sierra Leone where knowledge about cardiovascular disease risk factors and costs were identified barriers to accessing care [47]. The lower ratings of health system quality outcomes including trust and satisfaction (met need) among people not seeking recent care was consistent with work from the Lancet HQSS Commission highlighting their importance in achieving the quality needed for effective, people centered care [1]. More work to understand the scope and causes of this challenge in similar settings is needed to develop effective interventions to strengthen the quality and experience of primary and secondary care to ensure not just once-off access but continuity and comprehensiveness of care, core dimensions of effective primary care [48, 49].

Shared decision making is defined as "a process jointly shared by patients and their health care provider”. In our study, SUDM was found to be the most consistent factor associated with higher health system quality outcomes including satisfaction, confidence in the health systems, and health system quality outcomes. It aims at helping patients play an active role in decisions concerning their health, which is the ultimate goal of patient-centered care [50]. Shared decision making has been studied since the 1990’s and seen as increasingly important as the push for more people-centered primary care has emerged from the World Health Organizations and the Astana Declaration in 2018 [51]. The importance of shared decision making and effective communication for management of chronic conditions has been a focus of research in high income countries with lower rates of shared decision making being found among older individuals and those with poorer health, and associated with lower adherence to care and treatment [20, 21]. Achieving shared decision making requires engagement in decision making, effective communication, and good provider-patient relationships, factors which were captured in our SUDM measure. Similarly to our study, higher rates of shared decision making have been associated with better satisfaction, identifying an area for improving quality and outcomes of care for older individuals and people with NCDs [52].

Rating of care experience variables again pointed to areas where change is needed. Participants reported high ratings of some areas of visit experience (ability to follow advice and trust in provider skills), while other areas were lower, with one-half or fewer reporting high provider technical skills, clarity of communication, or involvement in decision making. Compared with other studies, clarity of communications was lower in our study (48% versus 66–100% in Tanzania and close to 60% in Ghana), although variability in populations, survey questions, and scoring makes comparisons challenging [20, 53]. In contrast, in Ghana female patients gave lower ratings for involvement, although the population was younger overall than in our study [19].

Overall trust and confidence in the health system was high, but lower among those not seeking recent care, who also reported lower met needs during their last care encounter. In another study in Burkina Faso, perceived quality of care was a determinant for retention in care, which is important for the continuity needed for NCDs and effective primary care more broadly, and identifying an area where improvement is needed [54]. This evidence for the relationship between uptake, retention, process, and outcomes of care experience offer a potential opportunity for improving continuity for the aging population and growing numbers of people with chronic conditions.

While geographic access was only rarely given as a reason for no recent care seeking, 14.5% had to borrow or sell something to attend a clinic, representing a significant burden among a population with high poverty. This measure also may underestimate cost burdens such as individuals who had to forgo consumption of other goods or services such as food to access their health care. While the lower wealth among non-users was similar to findings to another study, they also found higher rates of financial access as a barrier than in our study [55].

Our study had some key limitations. First, we were unable to collect all the dimensions of the traditional health systems responsiveness domains—aspects such as respect and confidentiality might have added to our understanding of care experience in this population. The self-reported nature of previous health information may have underestimated actual prevalence due to absent or forgotten diagnoses. We also limited our analyses to individuals visiting a public sector facility providing primary or secondary level care to focus on the local care system delivery, excluding the small proportion of participants using private or higher-level facilities. However, given the expanding role of the private sector in many countries, future work focusing on these facilities should be planned. We also did not collect the provider cadre who delivered the care, so can not comment on differences based on provider type. Finally, although statistically significant, the clinical significance was more limited for some variables where the odds ratio was close to one. One exception is the results for the SUDM variable which is measuring for every one-point increase in the variable, so the association increases when aggregated over multiple point changes.

In conclusion, we provide a comprehensive picture of public-sector health facility care seeking behaviors and user quality experiences among older individuals in rural Burkina Faso. A minority of individuals have sought recent care, most frequently for acute conditions, despite a burden of NCDs which need continuity of care. Among those with recent visits, the importance of shared understanding and engagement in decision making was seen across all measured health systems quality outcomes. Situating our findings was limited by the availability of comparable population-representative samples in rural, low-income settings–efforts to measure similar patient experiences should provide substantial benefit. Our findings provide insights into designing health system and care delivery interventions to improve the experience and involvement in care of the growing older population in rural LMICs. These interventions are particularly important for those with chronic conditions for whom ongoing care is critical to reducing preventable mortality and mortality.

## Supporting information

S1 Appendix (DOCX)

S1 Fig Consort diagram. (TIF)

S2 Fig Results from exploratory factor analysis. (TIF)

S1 File Research questionnaire. (DOCX)

S2 File Study tool. (PDF)
