# Correction: Older persons experiences of healthcare in rural Burkina Faso: Results of a cross sectional household survey

**DOI:** 10.1371/journal.pgph.0002510

**Published:** 2023-10-11

**Authors:** Ellen M. Goldberg, Mamadou Bountogo, Guy Harling, Till Baernighausen, Justine I. Davies, Lisa R. Hirschhorn

The last two authors, Justine I. Davies and Lisa R. Hirschhorn, should be noted as joint senior authors of this work.
