# Supplementary figures and images for: Older persons experiences of healthcare in rural Burkina Faso: Results of a cross sectional household survey
Source: PLOS Glob Public Health. 2022 Jun 9;2(6):e0000193. doi: 10.1371/journal.pgph.0000193 (PMC10021992; doi:10.1371/journal.pgph.0000193)

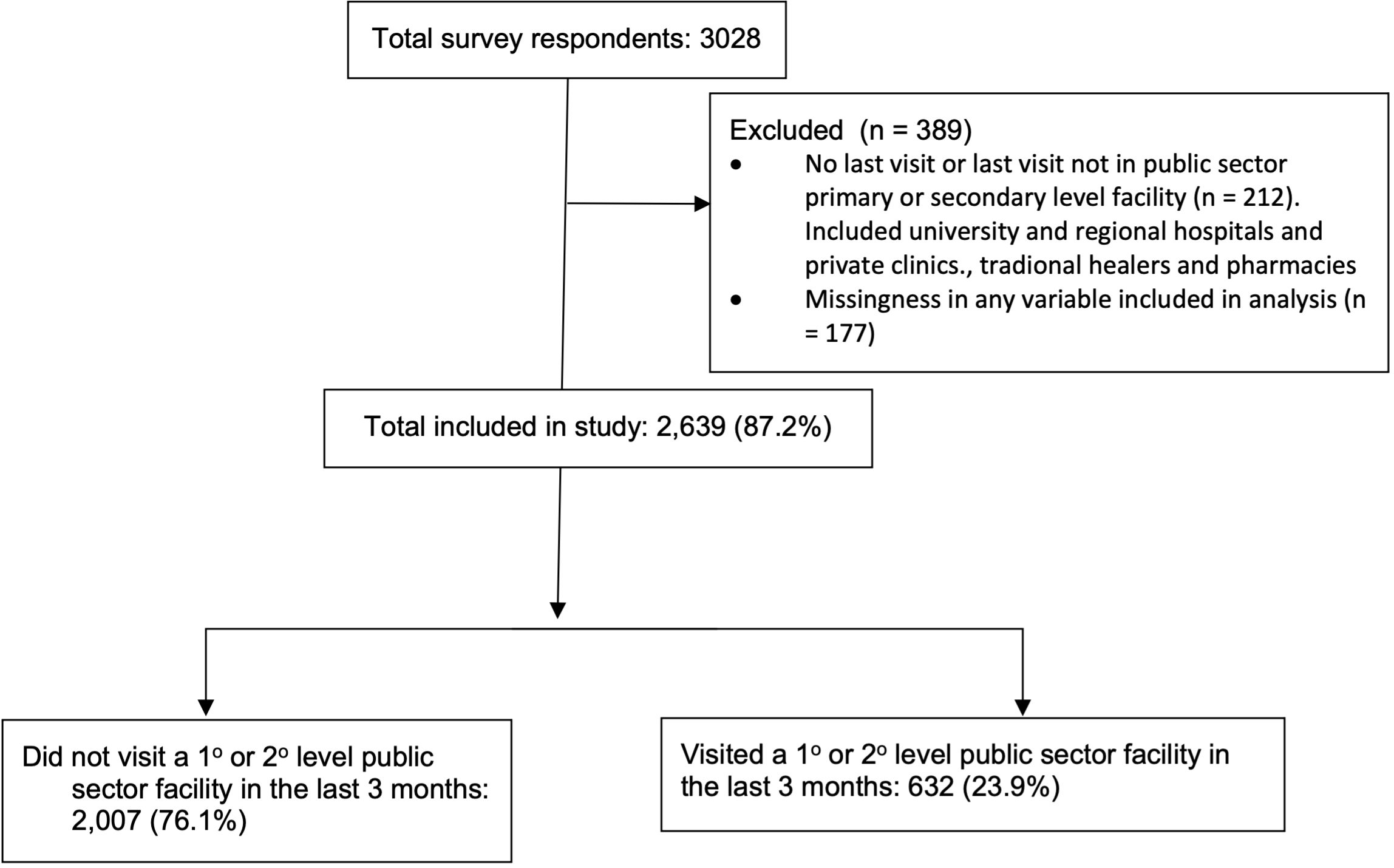

Supplement: S1 Fig — (TIF) [file pgph.0000193.s002.tif]

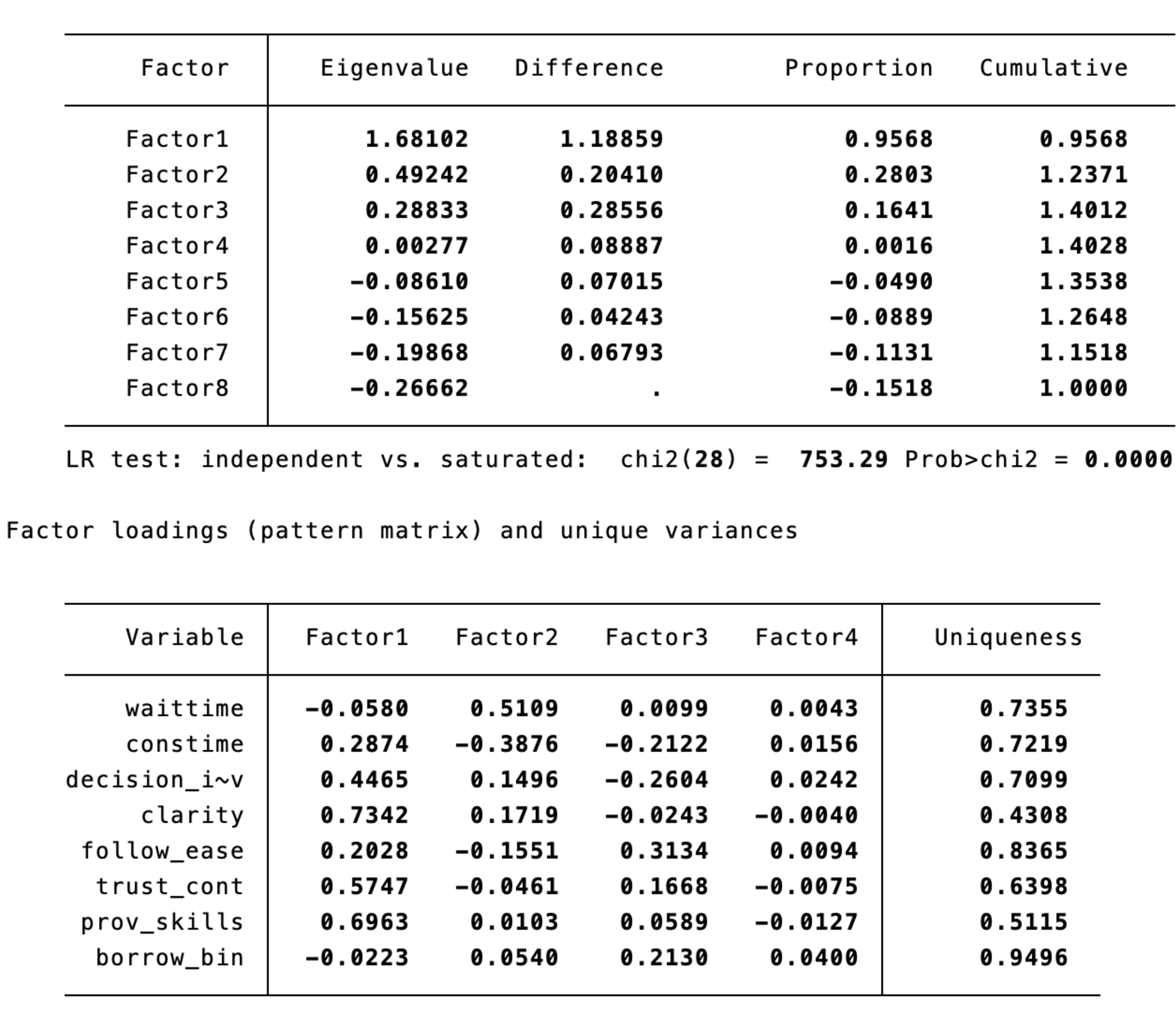

Supplement: S2 Fig — (TIF) [file pgph.0000193.s003.tif]
