# Supplementary material for: Older persons experiences of healthcare in rural Burkina Faso: Results of a cross sectional household survey
Source: PLOS Glob Public Health. 2022 Jun 9;2(6):e0000193. doi: 10.1371/journal.pgph.0000193 (PMC10021992; doi:10.1371/journal.pgph.0000193)
Supplement: S2 File — (PDF) [file pgph.0000193.s005.pdf]

Nouna Aging Pilot: Survey Instrument 1.0

Date: |__|__|/|__|__|/|__|__|__|__| Day / Month/Year

Household ID number |__|__|__|__|

Interviewer ID number |__|__|__|__|

Supervisor ID number |__|__|__|__|

Respondent ID number |__|__|__|__|

Time at start of interview: |__|__|:|__|__| Hours : Minutes

Section I: Sociodemographic Characteristics

A. Demographic Information, household composition and educational attainment

DEMOGRAPHIC INFORMATION		
D1	Are you the head of this household?	Yes…………..…………………………1<br>No……………………………………..2
D2	What is your relationship to the head of this household?	Partner<br>Parent<br>Grandparent<br>Grandson or Granddaughter<br>Son or daughter<br>Aunt or uncle<br>Niece or nephew<br>Cousin<br>Tenant<br>Friend<br>Other, specify: _________________
D3	Sex. Ask if not clear:	Male……………………………………..1<br>Female……………………………………2
D4	How old are you?	__________ years
D5	How many people over the age of 18, including yourself, live in your house-hold?	__________ people
D6	How many people under the age of 18 live in your household?	__________ people
D7	In total, how many years have you spent at school or in full-time study?	__________ years
D8	What is the highest level of education you have completed?	No Formal Schooling………….1<br>Less than Primary……………….2<br>Primary complete………………3<br>Some secondary ………………..4<br>Secondary complete………….5<br>High School complete………..6<br>College/University……………..7
D9	What is your marital status?	Never Married……………………….1<br>Currently Married…………………2<br>Separated……………………………..3<br>Divorced……………………………….4<br>Widowed……………………………….5<br>Cohabitating………………………….6<br>Refused………………………………….7

B. Socioeconomic Status, Health Expenditures, and Insurance

B1. Household assets

HOUSEHOLD ASSETS		
W1	What is the main source of water drunk by members of your household?	• Eau du robinet dans le logement<br>• Eau du robinet dans la cour/concession<br>• Eau du robinet public/borne fontaine<br>• Eau du robinet chez le voisin<br>• Puits à pompe ou forage<br>• Puits protégés<br>• Puits non protégés<br>• Source protégée<br>• Source non protégée<br>• Eau de pluie<br>• Camion citerne<br>• Charette avec petite citerne/tonneau<br>• Eau de surface<br>• Eau en bouteille<br>• Autre
W2	How long does it take to get the water and return home?	• Time, in minutes
W3	What type of toilet do members of your household usually use?	• Chasse d'eau - à un système d'égout<br>• Chasse d'eau - à un système d'égout - shared<br>• Chasse d'eau - à une fosse septique<br>• Chasse d'eau - à une fosse septique - shared<br>• Chasse d'eau - à des latrines<br>• Fosses/latrines - ventilées ameliorées (VIP)<br>• Fosses/latrines - ventilées ameliorées (VIP) - shared<br>• Fosses/latrines - avec dalles<br>• Fosses/latrines - avec dalles - shared<br>• Fosses/latrines - sans dalles/trou ouvert<br>• Fosses/latrines - sans dalles/trou ouvert - shared<br>• Toilettes à compostage<br>• Toilettes à compostage - shared<br>• Pas de toilette/nature<br>• Autre<br>• Autre - shared
W4	Do you share this toilet with other households?	• Yes/No
W5	What type of fule does your household usually use for cook-ing?	• Gaz propane liquifié (GPL)<br>• Charbon de bois<br>• Bois<br>• Repas non préparé dans le mémage<br>• Autre
W6	Do you have a separate room that you use as a kitchen?	• Yes/No
W7	How many bedrooms does this household have?	• Number
W8	Does your household own livestock, herds, other farm ani-mals or poultry?	• Yes/No, if N, skip next questions if No

W9	Which of the following animals does your household own:	• Yes/No
W10	Vaches laitières ou taureaux ?	• Yes/No
W11	Autre bétail ?	• Yes/No
W12	Chevaux, ânes ou mules ?	• Yes/No
W13	Chèvres ?	• Yes/No
W14	Moutons ?	• Yes/No
W15	Poulets ou autre volaille ?	• Yes/No
W16	Does anyone in your household own farmland?	• Yes/No, skip next question if No
W17	How many hectares of farmland do household members have?	• Number
	In this household, doyou have :	
W18	L'électricité ?	• Yes/No
W19	Un poste radio ?	• Yes/No
W20	Une télévision ?	• Yes/No
W21	Un téléphone fixe ?	• Yes/No
W22	Un ordinateur ?	• Yes/No
W23	Un réfrigérateur ?	• Yes/No
W24	Un table?	• Yes/No
W25	Des chaises?	• Yes/No
W26	Une Armoire/bibliothèque?	• Yes/No
	Does a member of this household own :	
W27	Une montre ?	• Yes/No
W28	Un téléphone portable ?	• Yes/No
W29	If yes, how many mobile phones in your household are currently in use?	• Number
W30	How many of the mobile phones currently in use in your household are “smartphones”?	• Number
W31	If so, do you pay for mobile data for any of these phones?	• Yes/No
W32	Une bicyclette ?	• Yes/No
W33	Une motocyclette ou un scooter ?	• Yes/No
W34	Une charrette tirée par un animal ?	• Yes/No
W35	Une voiture ou une camionnette ?	• Yes/No
W37	Does any household member have a bank account ?	• Yes/No

B2. Short Consumption/Health Expenditure

I would like to ask you some questions about how much your household spends on health services and other things.

For all questions in this section report all values in local currency, whether paid in cash or in kind.

1. In the last 4 weeks, how much did your household spend in total?

2. In the last 4 weeks, how much did your household spend on:
   a. Food, including such things as [rice], meat, fruits, vegetables, and cooking oils. Include the value of any food that was produced and consumed by the household, and exclude alcohol, tobacco and restaurant meals.
   b. Housing, gas, electricity, water, telephone, and heating fuel
   c. Education fees and supplies
   d. Health care costs, excluding any insurance reimbursements
   e. Voluntary insurance premiums or prepaid health plans
   f. All other goods and services

3. I would like to ask you more specific questions about how much your household spent on health services. When answering these questions, think about all of the times that any household member used a health service in the last 4 weeks. Please exclude costs to be reimbursed by insurance and any transportation costs. In the last 4 weeks, how much did your household spend on:
   a. Care that required staying overnight in a hospital or health facility
   b. Care by doctors, nurses, or trained midwives that did not require an overnight stay
   c. Care by traditional or alternative healers
   d. Dentists
   e. Medication or drugs
   f. Health care products such prescription glasses, hearing aids, prosthetic devices, etc.
   g. Diagnostic and laboratory tests such as X-rays or Blood tests
   h. Any other health care products or services that were not included above

4. In the last 12 months, how many times did members of your household go to a hospital and stay overnight (ENTER NUMBER OF TIMES FOR ALL HOUSEHOLD MEMBERS IN TOTAL. IF NONE, ENTER "0" and skip next question)

5. In the last 12 months, how much did the household pay for all costs associated with overnight stays in a hospital? Please exclude any expenses in the last 4 weeks that you have already told me about, and exclude any reimbursements from insurance.

B3. Health insurance

I would like to ask you some questions about health insurance. When we say someone is "covered by health insurance", we mean that he or she is enrolled with an organization that pays for health care costs if he or she gets sick or injured.

1. Are any of the members of this household covered by a community based insurance plan?
   a. Yes
   b. No
2. If Yes: Are all the members of this household covered by health insurance?

   a. Yes
   b. No
3. If No to 2: How many of this household's members under the age of 18 are covered by health insurance?
   a. Yes
   b. No
4. If No to 2: How many of this household's members over the age of 18 are covered by health insurance?
   a. Yes
   b. No

B4. LSMS Consumption

B4 will be asked to a randomly selected 1600 individuals. Within this group, 400 individuals will be randomized to each of four arms B4a-B4d.

1. What type of housing arrangement do you have?
   a. Owner with title
   b. Owner without title
   c. Tenant
   d. Employer provided housing
   e. Free housing
   g. Homeless or temporary housing
   h. Other, specify

2. How much is your monthly rent (if you rent) or do you think your rent would be (if you do not)?

For each of the list items, ask the following questions (drop from B1 wealth questionnaire as appropriate):

1. Does a household member have [ARTICLE] in good working order?
2. What is the number of [ARTICLE]?
3. How many years have you been in possession of? (if several, consider the age of the last)
4. What is the purchase value of the ARTICLE? (If several, consider the last one)
5. What is the current value (or selling price) of the ARTICLE? (If several, consider the last one)

Auto, Motorcycle, Bicycle, Sunscreen, TV Set, VCR/DVD, Radio, Stereo, Computer, Antenna with Cable Box, Mobile Phone, Landline Phone, Refrigerator, Freezer, Stove (Gas, Electric), Fireplace, Electric Iron , Charcoal iron, Fan, Air conditioning, Generator, Complete dining table, Bed, Mattress, Complete lounge, Buffet

For each of the item listed below, ask the following questions:

1. How much has the household spent on this product in the past 7 days?
2. Has the household consumed this item in the past 7 days? (if no, skip to question 5)
3. If Yes, on how many days out of the last 7 days did you consume this product?
4. Quantity and value of the item consumed coming from purchases in the past 7 days? (standard codes)
5. Quantity and value of the item consumed coming from own production in the past 7 days? (standard codes)
6. Quantity and value of the item consumed coming from gifts or in return for work/barter in the past 7 days? (standard codes)

- Rice; Corn; Sorghum; Fonio; Small millet; Corn flour; small millet flour; Sorghum flour; Wheat flour; Bread; Pasta; Other cereals; Other products based on cereals
- Fresh beef; Fresh sheep/goat; Pork; Fresh poultry; Dry fish; Fresh fish; Smoked fish; Other meat/fish
- Milk; Dairy products; Eggs
- Oils; Shea butter; Peanut paste; Other oil/fat
- Yams; Potato; Cassava; Okra; Kapok; Onions; Fresh tomatoes; Tomato paste; Leaves; Beans; Aubergine, Carotte, Chou, Haricots verts, Pomme de terre, Concombre, Tomate, Laitue, Petits pois, Other veg/tubers
- Ananas, Avocat, Banane, Goyave, Mangue, Orange, Papaye, Pastèque, Pomme ; other fruits
- Sugar cubes; Sugar power
- Salt; Stock cubes; Soumala
- Coffee; Tea; Other mass-produced non-alcoholic drinks (including sugar sweetened bevarages); Mineral water
- Traditional beer; Imported beer; Local beer; Wine/liquor; Cola nuts; Other narcotics
- Cigarettes; Chewing/local tobacco

For each of the items listed below, ask the following questions:

1. Has your household bought or received as gifts this item in the past 3 months? (if no, skip to next item)
2. How much has your household spent on buying this item in the past 3 months?
3. What is the total value of this item received as a gift, for work or in barter, in the past 3 months?

*Time period varies for those topics marked **
B4a. 8 health items, period for health items 1 month, except hospital services (3 months)
B4b. 4 health items, period for health items 1 month, except hospital services (3 months)
B4c. 8 health items, period for health items 3 months, except hospital services (12 months)
B4d. 4 health items, period for health items 3 months, except hospital services (12 months)

- Clothing fabric; Men's outerwear; Men's underwear; Women's outerwear; Women's underwear; Children's clothing; School uniform; Other clothing/accessories; Making/repairing men's clothing; Making/repairing women's clothing; Cleaning clothing; Men's shoes; Women's shoes; Children's shoes; Repair/rental of footwear
- Water (can/bucket/basin/barrel); Firewood; Charcoal; Electricity bill; Gas; Rental payments; Water bill; Garbage collection; Bricks (for repair); Imported cement; Local cement; Iron concrete; Sand; Pain; Septic tank drainage; Other domestic services/costs
- Electric battery; Insecticide spray; Tea towels; Domestic employees; Housekeeping; other household services; bed/mattresses; wardrobe/sideboard; chairs; dining room; other room; Mats; Flooring; Mosquito nets; Household textiles (curtains, towels); Refrigerators/freezers; Washing & drying machine; Gas/electric cooker; fireplace; heaters/humidifiers; vacuum cleaners; sewing/knitting machines; mixers; Irons (pressing); dishwashers; microwaves; fans; other household appliances; Glasses/crockery/utensils; major tools/equipment; small tools/accessories
- * B4a or B4c. Modern medicines; traditional medicines; other medical products; therapeutic materials; medical/dental services; lab/radiology services; medical assistant services; hospital services
- * B4b or B4d. Medicines; tests/diagnostics (incl. lab/radiology); consultations/provider fees; hospital services.
- Bus; taxi; other transport; parts/accessories; maintenance/repair of bikes/vehicles; other transport costs; new cars; new motorcycles; new bikes; second-hand car; second-hand motorbike; carts; petrol; motor oil; lubricants
- Telephone credit; postage; internet access; other phone services; other equipment

- Games/hobbies; sports equipment; garden supplies; pets incl. food; sport/cinema/culture fees; CD/video/DVD; gambling; other recreation; newspapers; books etc; holidays; TV; CD/DVD players portable and not; TV antennae; radio/audio equipment; hi-fi channel; cameras etc; computers; musical instruments; gaming consols; other durable leisure goods; repair of leisure goods
- School tuition (primary, secondary, tertiary, other)
- Restaurants; canteen food; Hotels/other lodging
- Milling of cereals; Milling of tubers; hair salon; jewelry/watches/clocks; other travel; social welfare/day care; home insurance; health insurance; travel/fire insurance; other non-life insurance; bank/financial charges; funeral costs

Section II: Physical Health

A. ADLs (WHODAS 2.0, ADLS)

WHODAS 2.0		
	In the past 30 days, how much difficulty did you have in:	
A1	Standing for long periods such as 30 minutes?	None……………………………………….1<br>Mild………………………………………..2<br>Moderate……………………………….3<br>Severe…………………………………….4<br>Extreme/cannot do…………………5
A2	Taking care of your household responsibilities?	None……………………………………….1<br>Mild………………………………………..2<br>Moderate……………………………….3<br>Severe…………………………………….4<br>Extreme/cannot do…………………5
A3	Learning a new task, for example learning how to get to a new place?	None……………………………………….1<br>Mild………………………………………..2<br>Moderate……………………………….3<br>Severe…………………………………….4<br>Extreme/cannot do…………………5
A4	Joining in community activities (for example festivities, religious or other activities) in the same way as anyone else can?	None……………………………………….1<br>Mild………………………………………..2<br>Moderate……………………………….3<br>Severe…………………………………….4<br>Extreme/cannot do…………………5
A5	How much have you been emotionally affected by your health problems?	None……………………………………….1<br>Mild………………………………………..2<br>Moderate……………………………….3<br>Severe…………………………………….4<br>Extreme/cannot do…………………5
A6	Concentrating on doing something for 10 minutes?	None……………………………………….1<br>Mild………………………………………..2<br>Moderate……………………………….3<br>Severe…………………………………….4<br>Extreme/cannot do…………………5
A7	Walking a long distance such as a kilometer?	None……………………………………….1<br>Mild………………………………………..2<br>Moderate……………………………….3<br>Severe…………………………………….4<br>Extreme/cannot do…………………5
A8	Dealing with people you do not know?	None……………………………………….1<br>Mild………………………………………..2<br>Moderate……………………………….3<br>Severe…………………………………….4<br>Extreme/cannot do…………………5
A9	Maintaining a friendship?	None……………………………………….1

		Mild……………………………………….2<br>Moderate………………………………3<br>Severe…………………………………….4<br>Extreme/cannot do…………………5
A10	Your day to day work or duties?	None……………………………………..1<br>Mild……………………………………….2<br>Moderate………………………………3<br>Severe…………………………………….4<br>Extreme/cannot do…………………5

Basic ADLs		
	We need to understand difficulties people may have with various activities because of a health or physical problem. Please tell me whether you have difficulty performing any of the following tasks on a regular basis. Exclude any difficulties that you expect to last less than three months.	
B1	Because of a health or memory problem do you have any difficulty with walking across a room?	No difficulty…………………………..1<br>Some difficulty……………………….2<br>Can't do, and want to do………..3<br>Can't do, but don't need to……..4
B2	Do you ever use equipment or devices (e.g., cane) when crossing a room or walking elsewhere?	Yes...1<br>No...2
B3	Does anyone ever help you get across a room?	Yes...1<br>No...2
B4	If you have difficulty crossing a room, do you feel you get enough help, or do you need more help?	I get no help……………………………..1<br>I get some help, but not enough..2<br>I get enough help……………………..3
B5<br>*WHODAS	Because of health and memory problems, do you have any difficulty with dressing? Dressing includes taking clothes out, putting them on, buttoning up, and fastening a belt.	None……………………………………..1<br>(IF NONE, SKIP to B8)<br>Mild……………………………………….2<br>Moderate………………………………3<br>Severe…………………………………….4<br>Extreme/cannot do…………………5<br>Don't want to…………………………6
B6	If you have difficulty dressing, do you feel you get enough help, or do you need more help?	I get no help……………………………..1<br>I get some help, but not enough..2<br>I get enough help……………………..3
B7<br>*WHODAS	Because of health and memory problems, do you have any difficulty with bathing or showering?	None……………………………………..1<br>(IF NONE, SKIP TO B10)<br>Mild……………………………………….2<br>Moderate………………………………3<br>Severe…………………………………….4<br>Extreme/cannot do…………………5<br>Don't want to…………………………6

B8	If you have difficulty bathing or showering, do you feel you get enough help, or do you need more help?	I get no help……………………………..1<br>I get some help, but not enough..2<br>I get enough help……………………..3
B9	Because of health and memory problems, do you have any difficulty with eating, such as cutting up your food? (Definition: By eating, we mean eating food by oneself when it is ready.)	None……………………………………..1<br>(IF NONE, SKIP to B12)<br>Mild………………………………………2<br>Moderate………………………………3<br>Severe……………………………………4<br>Extreme/cannot do…………………..5<br>Don't want to………………………….6
B10	If you have difficulty eating, do you feel you get enough help, or do you need more help?	I get no help……………………………..1<br>I get some help, but not enough..2<br>I get enough help……………………..3
B11	Do you have any difficulty with getting into or out of the place where you sleep (such as a bed or chair)?	None……………………………………..1<br>(IF NONE, SKIP to B14)<br>Mild………………………………………2<br>Moderate………………………………3<br>Severe……………………………………4<br>Extreme/cannot do…………………..5<br>Don't want to………………………….6
B12	If you have difficulty getting into or out of the place you sleep, do you feel you get enough help, or do you need more help?	I get no help……………………………..1<br>I get some help, but not enough..2<br>I get enough help……………………..3
B13	Because of health and memory problems, do you have any difficulties with using the toilet, including getting up and down?	None……………………………………..1<br>(IF NONE, SKIP to B16)<br>Mild………………………………………2<br>Moderate………………………………3<br>Severe……………………………………4<br>Extreme/cannot do…………………..5<br>Don't want to………………………….6
B14	If you have difficulty using the toilet, do you feel you get enough help, or do you need more help?	I get no help……………………………..1<br>I get some help, but not enough..2<br>I get enough help……………………..3
B15	Who most often helps you with walking, dressing, bathing, eating, getting out of bed, using the toilet? [CHOOSE UP TO 3 PEOPLE.]	Spouse……………………………………..1<br>Parent.……………………………………2<br>Parent in law…………………………..3<br>Son…………………………………………4<br>Daughter…………………………………5<br>Brother……………………………………6<br>Sister……………………………………….7<br>Brother in law………………………….8<br>Sister in law……………………………..9<br>Son in law……………………………….10<br>Daughter in law………………………11<br>Grandson.……………………………….12<br>Granddaughter………………………..13<br>Other relative…………………………..14<br>Paid helper………15<br>Volunteer or Employee of facility…16

		Other……………………………………….17<br>No one helped…………………………18

B. Health-related QOL

WHOQOL		
	I would like you to think about your health and quality of life over the last two weeks.	
H1	How would you rate your quality of life?	Very poor…………………………………1<br>Poor…………………………………………2<br>Neither good nor poor……………..3<br>Good…………………………………………4<br>Very good…………………………………5
H2	How satisfied are you with your health?	Very dissatisfied………………………1<br>Dissatisfied……………………………….2<br>Neither satisfied nor dissatisfied……….3<br>Satisfied……………………………………4<br>Very satisfied…………………………..5
H3	Do you have enough energy for everyday life?	Not at all………………………………….1<br>A little………………………………………2<br>Moderate…………………………………3<br>Mostly……………………………………….4<br>Completely………………………………5
H4	How satisfied are you with your ability to perform your daily living activities? [e.g. cooking, cleaning, work]	Very dissatisfied………………………1<br>Dissatisfied……………………………….2<br>Neither satisfied nor dissatisfied……….3<br>Satisfied……………………………………4<br>Very satisfied…………………………..5
H5	How satisfied are you with yourself?	Very dissatisfied………………………1<br>Dissatisfied……………………………….2<br>Neither satisfied nor dissatisfied……….3<br>Satisfied……………………………………4<br>Very satisfied…………………………..5
H6	How satisfied are you with your personal relationships?	Very dissatisfied………………………1<br>Dissatisfied……………………………….2<br>Neither satisfied nor dissatisfied……….3<br>Satisfied……………………………………4<br>Very satisfied…………………………..5
H7	Have you enough money to meet your needs?	Not at all………………………………….1<br>A little………………………………………2<br>Moderately………………………………3<br>Mostly……………………………………….4<br>Completely………………………………5
H8	How satisfied are your with the conditions of your living place?	Very dissatisfied………………………1<br>Dissatisfied……………………………….2<br>Neither satisfied nor dissatisfied……….3<br>Satisfied……………………………………4<br>Very satisfied…………………………..5

C. Frailty (For disability, see WHODAS 2.0 above)

FRIED FRAILTY + FALLS		
F1	Have you or those close to you noticed that you have lost weight or become thinner over the last year?	Yes…………………………………………1<br>No…………………………………………..2<br>(IF NO, SKIP TO F3)
F2	How much weight have you lost (in kg)?	1. Provide in kilograms
F3	Does your health now limit the kinds or amounts of vigorous activities you can do, like digging, fetching water from a well or splitting firewood?	Yes, limited a lot……………………1<br>Yes, limited a little………………..2<br>No………………………………………..3
F4	How has your overall health changed in the last 12 months?	Much better……………………………..1<br>Better……………………………………..2<br>The same………………………………..3<br>Worse……………………………………..4<br>Much worse…………………………….5
	Please tell me how often you have felt this way during the past week:	
F5	I felt that everything I did was an effort.	Rarely (<1 days)…………………………..1<br>Some of the time (1-2 days)………….2<br>Occasionally (3-4 days)…………………3<br>Most or all of the time (5-7 days)….4
F6	I could not get going.	Rarely (<1 days)…………………………..1<br>Some of the time (1-2 days)………….2<br>Occasionally (3-4 days)…………………3<br>Most or all of the time (5-7 days)….4
	Please consider your activity during a usual week. The rest of the questions will provide extra information on sedentary behaviour.	
F7	On a usual weekday, how many hours did you spend sitting or reclining (excluding sleep)? This may include time sitting on a chair or bench, visiting friends, reading, sitting in church, sitting down to watch television.	________________________Hours<br>________________________Minutes
F8	On a usual weekend day, how many hours did you spend sitting or reclining (excluding sleep)? This may include time sitting on a chair or bench, visiting friends, reading, sitting in church, sitting down to watch television.	________________________Hours<br>________________________Minutes

Section III: Cognition & Mental Health (19 questions)

A. Cognition (Cognitive Battery)

	CSI-D (8 questions)	
C1	Now I am going to tell you three words and I would like you to repeat them after me:<br>Cart<br>House<br>Fish	[Iwer: Repeat the three words, up to a maximum of six times or until the person has remembered them all correctly. Then say: Very good, now try to remember these words because I will be asking you later.]
C2	[Iwer points to their elbow]<br>What do we call this?	Correct…………………………………1<br>Incorrect………………………………2
C3	What do you do with a hammer?<br>*Acceptable answer 'To drive a nail into something'*	Correct…………………………………1<br>Incorrect………………………………2
C4	Where is the local market/local store?	Correct…………………………………1<br>Incorrect………………………………2
C5	What day of the week is it?	Correct…………………………………1<br>Incorrect………………………………2
C6	What is the season?	Correct…………………………………1<br>Incorrect………………………………2
C7	Please point first to the window and then to the door	Correct…………………………………1<br>Incorrect………………………………2
C8	Do you remember the three words I told you a few minutes ago?	
	Cart	Correct…………………………………1<br>Incorrect………………………………2
	House	Correct…………………………………1<br>Incorrect………………………………2
	Fish	Correct…………………………………1<br>Incorrect………………………………2

B. Anxiety (GAD-2) & Depression (PHQ-9)

GAD-2 (2 questions)		
	Over the last 2 weeks, how often have you been bothered by the following problems?	
G1	Feeling nervous, anxious, or on edge	Not at all …………………………………0<br>Several days……………………………1<br>Over half the days……………………2<br>Nearly every day………………………3
G2	Not being able to stop or control worrying	Not at all …………………………………0<br>Several days……………………………1<br>Over half the days……………………2<br>Nearly every day………………………3

PHQ9 (9 questions)		
	Over the past two weeks, how often have you been bothered by any of the following problems?	
P1	Little interest or pleasure in doing things	Not at all…………………………………0<br>Several days……………………………1<br>More than half the days…………2<br>Nearly every day……………………3
P2	Feeling down, depressed or hopeless	Not at all…………………………………0<br>Several days……………………………1<br>More than half the days…………2<br>Nearly every day……………………3
P3	Trouble falling asleep, staying asleep, or sleeping too much	Not at all…………………………………0<br>Several days……………………………1<br>More than half the days…………2<br>Nearly every day……………………3
P4	Feeling tired or having little energy	Not at all…………………………………0<br>Several days……………………………1<br>More than half the days…………2<br>Nearly every day……………………3
P5	Poor appetite or overeating	Not at all…………………………………0<br>Several days……………………………1<br>More than half the days…………2<br>Nearly every day……………………3
P6	Feeling bad about yourself – or that you are a failure or have let yourself or your family down	Not at all…………………………………0<br>Several days……………………………1<br>More than half the days…………2<br>Nearly every day……………………3
P7	Trouble concentrating on things	Not at all…………………………………0<br>Several days……………………………1<br>More than half the days…………2<br>Nearly every day……………………3
P8	Moving or speaking so slowly that other people could have noticed. Or the opposite – becoming so fidgety or restless that you have been moving around a lot more than usual	Not at all…………………………………0<br>Several days……………………………1<br>More than half the days…………2<br>Nearly every day……………………3
P9	Thoughts that you would be better off dead, or thoughts of hurting yourself in some way	Not at all…………………………………0<br>Several days……………………………1<br>More than half the days…………2<br>Nearly every day……………………3

C. Subjective Expectations

INTERVIEWER: Recount the number of peanuts and check that you have 10 peanuts in the plate [__]. As you provide the explanation below, add the peanuts into the plate to illustrate what you say.

"I will ask you several questions about the chance or likelihood that certain events are going to happen. There are 10 peanuts in the cup. I would like you to choose some peanuts out of these 10 peanuts and put them in the plate to express what you think the likelihood or chance is of a specific event happening. One peanut represents one chance out of 10. If you do not put any peanuts in the plate, it means you think that the event will definitely NOT happen. As you add peanuts, it means that you think the likelihood that the event happens increases. For example, if you put one or two peanuts, it means you think the event is not likely to happen but it is still possible. If you pick 5 peanuts, it means that it is just as likely it happens as it does not happen (fifty-fifty). If you pick 6 peanuts, it means the event is slightly more likely to happen than not to happen. If you put 10 peanuts in the plate, it means you think the event will definitely happen. There are no right or wrong answers, I just want to know what you think.

INTERVIEWER: Report for each question the NUMBER OF PEANUTS put in the PLATE. After each question, replace the peanuts in the cup (unless otherwise noted). Interviewer: Remind respondent that he/she can put half a peanut if respondent wants to pick value between two whole peanuts (e.g., respondent thinks 1 and half peanuts (1.5) is the best answer). If respondent is not able to break the peanut in half, help him/her with this.

If respondent puts 10 (or 0) peanuts in question X1, prompt "Are you sure that this event will almost surely (not) happen?" Record if you prompted the respondent, and report the final answer only. Do NOT prompt for the remaining questions.

	Pick the number of peanuts that reflects how likely you think it is that...	# of peanuts in plate	Prompt for 0 or 10?
X1	*A person of your sex and age in your community will die within 5 years.*<br>*[Start with 10 peanuts and empty plate]*		
X2a	*you will die within a five-year period beginning today*<br>*([Start with 10 peanuts and empty plate. LEAVE PEANUTS ON PLATE)*		
X2b	*you will die within a ten-year period beginning today*<br>*do not remove peanuts from plate after respondent answered X2a, use the remaining peanuts; (IT IS POSSIBLE TO ADD ZERO ADDITIONAL PEANUTS)*		
X4	*a child born in this community will die before age 1*<br>*[Start with empty plate and 10 peanuts]*		
X5	*a child born in this community will die before age 5*<br>*[Start with empty plate and 10 peanuts]*		
X6	*you will have malaria within a five-year period beginning today*<br>*[Start with empty plate and 10 peanuts]*		
X7	*you will be hypertensive within a five-year period beginning today*<br>*[Start with empty plate and 10 peanuts]*		
X8	*you will be diabetic within a five-year period beginning today*<br>*[Start with empty plate and 10 peanuts]*		
X9	*you will experience illness or disability within a five-year period beginning today that will prevent you from doing your usual daily activities or work*<br>*[Start with empty plate and 10 peanuts]*		
X10	*you will need to be admitted to the hospital within the next year*<br>*[Start with empty plate and 10 peanuts]*		

	Pick the number of peanuts that reflects how likely you think it is that...	# of peanuts in plate	Prompt for 0 or 10?
X11	*you will have a large health expense within the next year that will require you to sell assets, borrow money, or reduce consumption*<br>*[Start with empty plate and 10 peanuts]*		
X12	*someone in this household will have a large health expense within the next year that will require the household to sell assets, borrow money, or reduce consumption[Start with empty plate and 10 peanuts]*		

D. Subjective aging

1. Many people feel older or younger than they actually are. How old do you feel most of the time?
   a. ______(years)

INSTRUCTIONS: The next questions are statements on how a person may feel about getting older. We are interested in how **YOU** feel about getting older and what you can tell us about your own experiences. We are mostly interested in whether certain experiences apply to you and to what extent you can relate to them given that you may have experienced them yourself as the years have passed.Please read each statement carefully and answer with one of “not at all”, “a little bit”, “moderately”, “quite a bit” or “very much”, depending on how much the statement reflects your own experience. There are no “right” or “wrong” answers to these statements. We are simply interested in your own personal experiences and your honest opinion.

	**With my increasing age, I realize that …**	**Not at all**<br>**1**	**A little bit**<br>**2**	**Moderately**<br>**3**	**Quite a bit**<br>**4**	**Very much**<br>**5**
2	…I appreciate relationships and people much more.					
3	…I pay more attention to my health.					
4	…my mental capacity is declining.					
5	…I have more experience and knowledge to evaluate things and people.					
6	…I have a better sense of what is important to me.					
7	…I have to limit my activities.					
8	…I have less energy.					
9	…I feel more dependent on the help of others.					
10	…I have more freedom to live my days the way I want.					
11	…I find it harder to motivate myself.					

INSTRUCTIONS: The next questions are statements that you may agree or disagree with.Please indicate your agreement with each item on a scale from 1 to 7, where 1 is “strongly disagree”, 4 is “neither agree nor disagree” and 7 is “strongly agree”.

		1	2	3	4	5	6	7
12	In most ways my life is close to ideal.							
13	I am satisfied with the current state of affairs in my life.							
14	If I could live my life over, I would change almost nothing.							
15	My life *does not* live up to the standards I have for a good life.							
16	I am satisfied with my life.							

E. Relationship satisfaction

IF Currently married or cohabiting: skip to Q2

1. Are you currently in a romantic relationship with someone?<br>For example: currently dating, being engaged, being married	Yes	No

IF No: Skip to next section

	Extremely unhappy	Fairly un-happy	A little unhappy	Happy	Very happy	Extremely happy
2. Please indicate the degree of happiness, all things considered, of your relationship.						
	Not at all	A little	Somewhat	Mostly	Almost completely	Completely
3. How true is the statement: “I have a warm and comfortable relationship with my partner”						
4. How rewarding is your relationship with your partner?						
5. In general, how satisfied are you with your relationship?						

<u>Section IV: Healthcare Utilization</u>

A. Medical History

MEDICAL HISTORY & STEPS		
MH1a	Have you ever had your blood pressure measured by a doctor or other health worker?	Yes……………………………………..1<br>No………………………………………2<br>Don't Know………………………...3
MH1b	Have you ever been told by a doctor or other health worker that you have raised blood pressure or hypertension?	Yes……………………………………..1<br>No………………………………………2<br>Don't Know………………………...3
MH1c	If yes, have you ever received treatment for raised blood pressure from a doctor or other health worker?	Yes……………………………………..1<br>No………………………………………2<br>Don't Know………………………...3
MH1d	If yes, during the past two weeks, have you been taking your treatment for raised blood pressure with medications prescribed by a doctor or other health worker?	Yes……………………………………..1<br>No………………………………………2<br>Don't Know………………………...3
MH2a	Have you ever had your blood sugar measured by a doctor or other health worker?	Yes……………………………………..1<br>No………………………………………2<br>Don't Know………………………...3
MH2b	Have you ever been told by a doctor or other health worker that you have raised blood sugar or diabetes?	Yes……………………………………..1<br>No………………………………………2<br>Don't Know………………………...3
MH2c	If yes, have you ever received treatment for raised blood sugar or diabetes from a doctor or other health worker?	Yes……………………………………..1<br>No………………………………………2<br>Don't Know………………………...3
MH2d	If yes, during the past two weeks, have you been taking your treatment for raised blood sugar or diabetes with medications prescribed by a doctor or other health worker?	Yes……………………………………..1<br>No………………………………………2<br>Don't Know………………………...3
MH3a	Have you ever had your cholesterol measured by a doctor or other health worker?	Yes……………………………………..1<br>No………………………………………2<br>Don't Know………………………...3
MH3b	Have you ever been told by a doctor, nurse, or other health worker that you have high cholesterol?	Yes……………………………………..1<br>No………………………………………2<br>Don't Know………………………...3
MH3c	If yes, have you ever received treatment for high cholesterol by a doctor or other health worker?	Yes……………………………………..1<br>No………………………………………2<br>Don't Know………………………...3
MH3d	If yes, during the past two weeks, have you been taking your treatment for high cholesterol with medications prescribed by a doctor or other health worker?	Yes……………………………………..1<br>No………………………………………2<br>Don't Know………………………...3
MH4a	Have you ever been told by a doctor or other health doctor that you have a heart disease (heart failure, heart attack, angina etc.)?	Yes……………………………………..1<br>No………………………………………2<br>Don't Know………………………...3

MH4b	If yes, have you ever received treatment for this heart problem?	Yes……………………………………..1<br>No……………………………………….2<br>Don't Know……………………….....3
MH4c	If yes, during the past two weeks, have you been taking your treatment for this heart problem?	Yes……………………………………..1<br>No……………………………………….2<br>Don't Know……………………….....3
MH5a	Have you ever been told by a doctor, nurse, or other healthcare worker that you have had a stroke?	Yes......................................1<br>No.......................................2
MH5b	Have you ever had sudden drooping of one side of your face?	Yes......................................1<br>No.......................................2
MH5c	Have you ever had sudden numbness, weakness or a dead feeling on one half of your body?	Yes......................................1<br>No.......................................2
MH5d	Have you ever suddenly had difficulty speaking or slurring of your speech?	Yes......................................1<br>No.......................................2
MH6a	Have you ever been told you have a chronic respiratory disease (COPD, asthma)?	Yes……………………………………..1<br>No……………………………………….2<br>Don't Know……………………….....3
MH6b	If yes, have you ever received treatment for this chronic respiratory problem?	Yes……………………………………..1<br>No……………………………………….2<br>Don't Know……………………….....3
MH6c	If yes, during the past two weeks, have you been taking your treatment for this chronic respiratory disease?	Yes……………………………………..1<br>No……………………………………….2<br>Don't Know……………………….....3
MH7a	Have you ever been told by a doctor that you have a cancer?	Yes……………………………………..1<br>No……………………………………….2<br>Don't Know……………………….....3
MH7b	If yes, have you ever received treatment for this cancer?	Yes……………………………………..1<br>No……………………………………….2<br>Don't Know……………………….....3
MH8a	Have you ever been told by a doctor, nurse, or other healthcare worker that you have tuberculosis?	Yes……………………………………..1<br>No……………………………………….2<br>Don't Know……………………….....3
MH8b	Have you been newly diagnosed with tuberculosis in the last 12 months?	Yes……………………………………..1<br>No……………………………………….2<br>Don't Know……………………….....3
MH8c	Have you ever received TB treatment prescribed by a doctor, nurse or other healthcare worker?	Yes……………………………………..1<br>No……………………………………….2<br>Don't Know……………………….....3
MH9a	Have you ever been tested for HIV?	Yes……………………………………..1<br>No……………………………………….2<br>Don't Know……………………….....3
MH9b	Have you ever tested positive for HIV?	Yes……………………………………..1<br>No……………………………………….2<br>Don't Know……………………….....3

MH10A	Do you have any of other health issue(s) that has lasted more than 3 months?	Yes……………………………………..1<br>No……………………………………….2
MH10b	If Yes: Please tell me what other health issue(s) you have that have lasted more than 3 months.	CODES FROM CRSN LIST OF CONDITIONS
MH11a	Have you ever been told by a doctor or other health worker that you should change your diet?	Yes……………………………………..1<br>No……………………………………….2<br>Don't Know………………………....3
MH11b	If Yes: Have you tried to change your diet after being told to?	Yes.....................................1<br>No......................................2
MH11c	Have you ever been told by a doctor or other health worker that you should do more exercise?	Yes……………………………………..1<br>No……………………………………….2<br>Don't Know………………………....3
MH11d	If Yes: Have you tried to do more exercise after being told to?	Yes.....................................1<br>No......................................2
MH12a	Have you ever been told by a doctor or other health worker that you should lose weight?	Yes……………………………………..1<br>No……………………………………….2<br>Don't Know………………………....3
MH12b	If Yes: Have you tried to lose weight after being told to?	Yes.....................................1<br>No......................................2
MH13a	In the last 12 months, have you had any event where you suffered from bodily injury?	Yes.....................................1<br>No......................................2
MH13b	If Yes: what was the cause of this injury?	Fall...1<br>Struck/hit by object.............2<br>Cut/stabbed.........................3<br>Gunshot.............................4<br>Fire/Heat Burn....................5<br>Drowning/near-drowning…6<br>Poisoning...........................7<br>Animal bite.........................8<br>Electric shock.....................9<br>Other:
MH13c	If Fall: what height did you fall from?	Fell from standing at ground level…1<br>Fell from higher than ground level..2
MH13d	If injury: did you suffer a physical disability as a result of being injured?	Yes...1<br>No..2

B. Medicines

MEDICINES		
	Thinking of all the medicines you take at the moment:	
M1	Do you ever forget to take your medicine?	Yes.....................................1<br>No......................................2
M2	Do you ever have problems remembering to take your medication?	Yes.....................................1<br>No......................................2

M3	When you feel better, do you sometimes stop taking your medicine?	Yes.....................................1<br>No......................................2
M4	Sometimes if you feel worse when you take your medicine, do you stop taking it?	Yes.....................................1<br>No......................................2
	Thinking of all the medicines you take, would you agree or disagree with the following statements:	
M5	I am convinced of the importance of my prescribed medication	Strongly agree<br>Slightly agree<br>Slightly disagree<br>Strongly disagree
M6	I worry that my prescribed medication will do more harm than good to me	Strongly agree<br>Slightly agree<br>Slightly disagree<br>Strongly disagree
M7	I feel financially burdened by my out-of-pocket expenses for my prescribed medication	Strongly agree<br>Slightly agree<br>Slightly disagree<br>Strongly disagree

C. Quality of Care and Satisfaction

QUALITY OF CARE		
Q2b	Where do you usually first go when you feel sick?	a. University Hospital Center (CHU)<br>b. Regional Hospital Center (CHR)<br>c. Medical Center with Surgical Antenna (CMA)<br>d. Medical Center (CM)<br>e. Center for Health and Social Promotion (CSPS)<br>f. Private clinic / care office<br>g. Private religious / NGO<br>h. Pharmacy<br>i. Community Health Worker<br>j. Traditional Healer<br>k. Other
Q1a	Have you visited a health facility **for yourself** in the past **4 weeks**?	Yes……………………………………..1<br>(IF YES, SKIP TO Q3)<br>No………………………………………2<br>Don't Know………………………...3
Q1b	Have you visited a health facility **for yourself** in the past **3 months**?	Yes……………………………………..1<br>(IF YES, SKIP TO Q3)<br>No………………………………………2<br>Don't Know………………………...3
Q2	Why have you not visited a health facility in the past 3 months? <u>SKIP to Q24</u>	Choose best matches from response<br>a. Was not sick<br>b. Preferred to see a healer<br>c. Preferred to see a community health worker<br>d. Institution not clean<br>e. Long wait<br>f. Unskilled staff/do not think staff are good

		g. Too expensive<br>h. No staff/closed institution<br>i. Ineffective treatment<br>j. Bad experience in the past<br>k. Too far away/could not get transport<br>l. Did not have time/childcare<br>m. Other, specify
Q3	For what reasons were you seeking care at your most recent visit? (mark all that apply	a. Fever / Malaria<br>b. Diarrhoea / stomach ache<br>c. Pain in the back, limbs or joints<br>d. Dental problem<br>e. Skin problem<br>f. Eye disease<br>g. High blood pressure<br>h. Other heart condition<br>i. Typhoid fever<br>j. Ear, Nose, Throat<br>k. Accident / Injury<br>l. Diabetes, including symptoms e.g. foot disease<br>m. Peptic ulcer<br>n. Meningitis<br>o. Breathlessness<br>p. Weakness of arm/legs<br>q. Antenatal care, post-natal care, or delivery<br>r. Family planning<br>s. Respiratory illness<br>t. Asthma<br>u. Malignant Tumor / Cancer<br>v. Other, specify
Q4	What type of health facility did you visit most recently?	a. University Hospital Center (CHU)<br>b. Regional Hospital Center (CHR)<br>c. Medical Center with Surgical Antenna (CMA)<br>d. Medical Center (CM)<br>e. Center for Health and Social Promotion (CSPS)<br>f. Private clinic / care office<br>g. Private religious / NGO<br>h. Pharmacy<br>i. Community Health Worker<br>j. Traditional Healer<br>k. Other
Q4b	What was the name of the facility?	Free text or could have the list of facilities and tick off whichever they say
Q4c	Who first consulted with you for this episode of illness?	a. Doctor (doctor)<br>b. Health attaché<br>c. Nurse (patented or state)<br>d. Pharmacist<br>e. Other modern health workers, facility based<br>f. Community Health Worker<br>g. Healer / Marabout<br>h. Other
	**Thinking about your most recent visit to a health care facility, I would like to ask you some questions about your experience**	

Q5	How long did you wait before your consultation?	______minutes or _____ hours
	**The next few questions are about how you felt about the last visit you made to a health facility.**	
Q6	What do you think about the care provider's medical knowledge and skills? (read out options)	Excellent...5<br>Very Good..4<br>Good..3<br>Fair..2<br>Poor..1<br>Don't Know.......................................88
Q7	How much time did you spend with the care provider (consultation time)?	______minutes
Q8	Did they advise you to come back?<br>(if no / don't know skip to Q11)	Yes...1<br>No..2<br>Don't Know...............................3
Q9	If yes, did they tell you why?	Yes...1<br>No..2<br>Don't Know...............................3
Q10	If yes, did you come back?	Yes...1<br>No..2<br>Don't Know...............................3
Q11	Did they refer you to another facility?<br>(if no / don't know skip to Q14)	Yes...1<br>No..2<br>Don't Know...............................3
Q12	If yes, did they tell you why?	Yes...1<br>No..2<br>Don't Know...............................3
Q13	If yes, did you go to this other facility?	Yes...1<br>No..2<br>Don't Know...............................3
Q14	Did they ask for further tests?<br>(if no / don't know skip to Q17)	Yes...1<br>No..2<br>Don't Know...............................3
Q15	If yes, did they tell you which ones and why?	Yes...1<br>No..2<br>Don't Know...............................3
Q16	If yes, did you make them?	Yes...1<br>No..2<br>Don't Know...............................3
Q17	What do you think about the provider's ability to explain things in a way that you could understand?	Excellent...5<br>Very Good..4<br>Good..3<br>Fair..2<br>Poor..1<br>Don't Know.......................................88
Q18	What do you think about your experience of being involved in making decisions for your treatment?	Excellent...5<br>Very Good..4

		Good…………………………………………..3<br>Fair..2<br>Poor...1<br>Don't Know......................................88
Q19	How much do you trust the skills and abilities of the health workers at this facility?	Very much..5<br>Quite a bit..4<br>Some…………………………………………..3<br>Very little..2<br>Not at all……………………………………1<br>Don't Know......................................88
Q20	How easy or difficult was it for you to follow the provider's advice?	Very Easy..5<br>Easy...4<br>fair…………………………………………..3<br>hard..2<br>very hard...1<br>Don't Know......................................88
Q21	Overall, thinking about your entire last visit, please rate how well the care you received met your health needs. That is, how much did the visit help solve your health problem or help you feel better?	Excellent..5<br>Very Good...4<br>Good…………………………………………..3<br>Fair..2<br>Poor...1<br>Don't Know......................................88
Q22	If you were prescribed medications, were you able to get it the same day as your visit	Yes……………………………………….1<br>No……………………………………….2<br>No-I was not prescribed a medication 3<br>Don't Know…………………….....4
Q23	Did you have to borrow money or sell anything to pay for this health care (including transportation, fees, medication)	Yes……………………………………….1<br>No……………………………………….2<br>Don't Know…………………….....3
Q24	How confident are you that if you became very sick tomorrow, you would be able to receive effective treatment from the health system?	Very confident……………4<br>Somewhat confident…..3<br>Not very confident………2<br>Not at all confident……..1<br>Don't know…………………88
Q25	Which of the following statements comes closest to expressing your overall view of the health care system in this country? (read all options out loud)	Our health care system has so much wrong with it that we need to completely rebuild it…….1<br>There are some good things in our health care system, but major changes are needed to make it work better….2<br>On the whole, the system works pretty well and only minor changes are necessary to make it work better….3

Section V: Physical Battery, Measurements & Laboratory Studies

Height/Weight		
	Height	
	Weight	
	Waist Circumference	
	Women: are you pregnant?	Yes……………………………………..1<br>No………………………………………2<br>Don't Know………………………...3

Blood Pressure		
	Blood Pressure Measurement 1	Systolic:<br>Diastolic:
	Blood Pressure Measurement 2	Systolic:<br>Diastolic:
	Blood Pressure Measurement 3	Systolic:<br>Diastolic:

Walk speed		
	Normal walk [REFER TO SHOWCARDS] Did respondent complete the walk at usual pace?	Yes…………………………………………………1<br>No, REFUSED………….……………………..2<br>No, cannot walk even with support...3
	Normal walk. Time at 4 meters:	______________________Seconds
	Normal walk: Second attempt	______________________Seconds

Grip strength		
	Have you had any surgery on your left arm, hand or wrist in the last 3 months OR arthritis or pain in your left hand or wrist?	YES...1<br>NO 2
	Have you had any surgery on your right arm, hand or wrist in the last 3 months OR arthritis or pain in your right hand or wrist?	Yes...1<br>No 2
	Which hand do you consider your dominant hand? [INSTRUCTIONS: IF A RESPONDENT IS AMBIDEXTROUS, THE HAND THAT IS USED FOR SIGNING/WRITING IS CONSIDERED THE DOMINANT HAND.]	LEFT…………………………………………1<br>RIGHT……………………………………..2<br>USE BOTH THE SAME…………………3
	Did respondent complete the grip strength test?	Yes<br>No, refused<br>No, unable to understand instructions

		No, too weak to perform test
	First test, left hand	___________________________Kg
	Second test, left hand	___________________________Kg
	First test, right hand	___________________________Kg
	Second test, right hand	___________________________Kg

Chair Rise		
	Single Chair Rise	
	A. Safe to Stand without Help	Yes……………………………………….1<br>No……………………………………….2
	B. Result	Stood without using arms……………..1<br>Used arms to stand……………………….2<br>Did not complete or unable………….3
	C. If failed, provide reason:	Tried but unable…………………1<br>Unable to stand unassisted…2<br>Not attempted, you felt unsafe…………..3<br>Not attempted, participant felt unsafe..4<br>Unable to understand instructions………5<br>Other (Specify):<br>Refused…………………………….7
	Repeated Chair Rise	
	A. Safe to Stand X 5	Yes……………………………………….1<br>No……………………………………….2
	B. Time to stand	1. Provide in seconds
	C. If did not attempt or failed, provide reason:	Tried but unable…………………1<br>Unable to stand unassisted…2<br>Not attempted, you felt unsafe…………..3<br>Not attempted, participant felt unsafe..4<br>Unable to understand instructions………5<br>Other (Specify):<br>Refused…………………………….7

Laboratory Studies		
	Venous Blood (via phlebotomy)	Completed………………………….1<br>Refused…………………………….2<br>Otherwise not completed……3

<u>Section VI: Value of statistical life</u> (Everyone randomized to get 1 of the 4 experiments A, B, C or D)

Let me briefly explain the way I would like us to approach the following question. We have come up with hypothetical situations that we would like you to imagine yourself in. You will be faced with difficult decisions that involve the risk of death. We are not in any way suggesting that by imagining yourself in these situations, you will experience what we present you with. We are also most certainly not wishing illness or even death upon you. We simply want to find out what choices people make when they find themselves in such difficult situations. Please simply ask yourself: what would I do if I was to find myself in such a situation? There are no right or wrong answers to the question we ask. Does that make sense?

Experiments A & B. Please imagine the following two situations. In the first situation, you live in a community in which **5 out of 100** people die every year: you are one of these 100 people, thus the chance that you will die is 5 out of 100. In the second situation, you live in a community in which **3 out of 100** people die every year: thus the chance that you will die is 3 out of 100. In both situations the death is sudden and painless. You should also imagine that except for the risk of death, everything in your life is the same in the two communities. The picture below helps to illustrate the risk. For each 100 people, the crossed-out figures will die. Imagine yourself to be one of the 100 people, but you do not yet know which one. Thus, you might be one of those who die, or one of those who do not die.

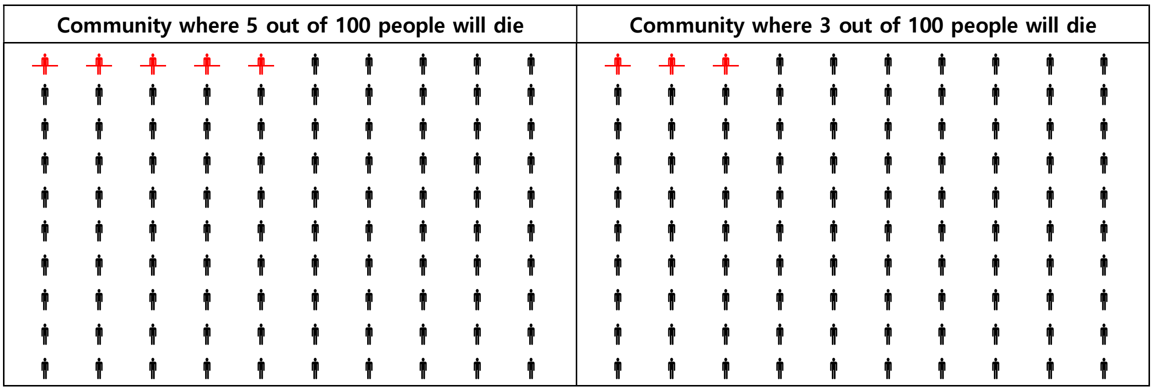

Q1. Imagine that you live in a community where the risk of death is 5 out of 100 people each year. You can now pay a certain amount of money each year to instead live in a community with a risk of death of 3 out of 100 people per year. Here we would like to know what the maximum annual fee in CFA is that you would be willing to reduce your risk of death from 5 out of 100 people to a risk of death of 3 out of 100 people. In other words, what would be the maximum <u>annual</u> fee in CFA that you would be willing to pay, to live in a community where the risk of death is 3 out of 100 people rather than 5 out of 100 people. Please be sure to take into account what you can <u>actually afford</u> to pay, and not if you had unlimited money. *[Start from low amounts going up, asking "Would you pay no more than X, or more?". If respondent indicates that his/her value is in between two values in the list, mark the two values bracketing the value indicated by respondent.]*

Experiment A

Amount (annual, CFA)	Check maximum value.
0	O
1,000	O
2,500	O
5,000	O
10,000	O
15,000	O
20,000	O
25,000	O
30,000	O
35,000	O
40,000	O
45,000	O
50,000	O
60,000	O
70,000	O
80,000	O
100,000	O
150,000	O
200,000	O
400,000	O

Experiment B

Amount (annual, CFA)	Check maximum value.
0	O
5,000	O
10,000	O
25,000	O
50,000	O
100,000	O
150,000	O
200,000	O
250,000	O
300,000	O
400,000	O
500,000	O
600,000	O
700,000	O
800,000	O
1,000,000	O
1,200,000	O
1,400,000	O
1,700,000	O
2,000,000	O

Experiments C & D. Please imagine the following two situations. In the first situation, you live in a community in which **3 out of 100** people die every year: you are one of these 100 people, thus the chance that you will die is 3 out of 100. In the second situation, you live in a community in which **5 out of 100** people die every year: thus the chance that you will die is 5 out of 100. In both situations the death is sudden and painless. You should also imagine that except for the risk of death, everything in your life is the same in the two communities. The picture below helps to illustrate the risk. For each 100 people, the crossed-out figures will die. Imagine yourself to be one of the 100 people, but you do not yet know which one. Thus, you might be one of those who die, or one of those who do not die.

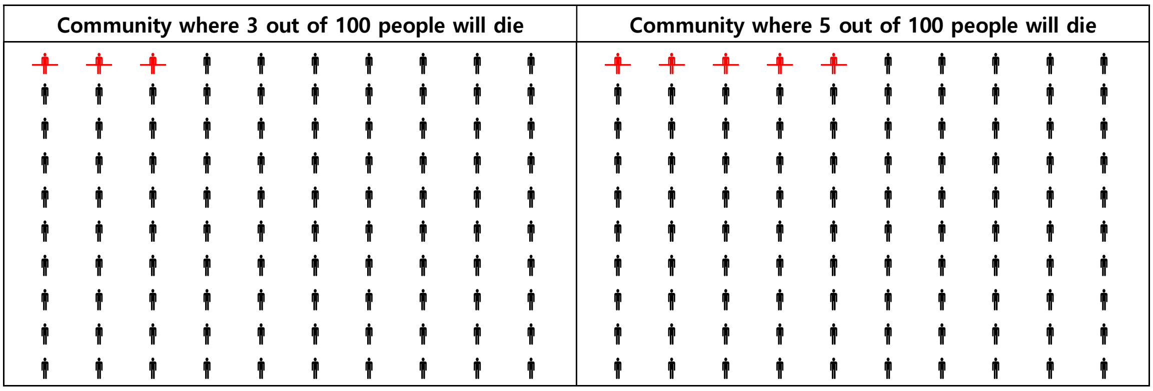

Q1. Imagine that you live in a community where the risk of death is 3 out of 100 people each year. You can now receive a certain amount of money each year if you accept to instead live in a community with a risk of death of 5 out of 100 people per year. Here we would like to know what the minimum annual fee in CFA is that you would require as compensation to accept your risk of death to increase from 3 out of 100 people to a risk of death of 5 out of 100 people. In other words, what would be the minimum annual fee in CFA that you would require as compensation, to accept living in a community where the risk of death is 5 out of 100 people rather than 3 out of 100 people. Please be sure to think about the absolute minimum compensation you would require, which may not be the amount someone else is able or willing to compensate you. That is, we want to know what your personal acceptable minimum is, irrespective of what you believe could be paid by someone. Please check the box for the minimum amount of CFA below. *[Start from low amounts going up, asking "Would accept the risk increase for X, or do you require more?" If respondent indicates that his/her value is in between two values in the list, tick the two value bracketing the value indicated by respondent.]*

Experiment C

Amount (annual, CFA fr)	Check minimum value.
0	O
1,000	O
2,500	O
5,000	O
10,000	O
15,000	O
20,000	O
25,000	O
30,000	O
35,000	O
40,000	O
45,000	O
50,000	O
60,000	O
70,000	O
80,000	O
100,000	O
150,000	O
200,000	O
400,000	O

Experiment D

Amount (annual, CFA fr)	Check minimum value.
0	O
5,000	O
10,000	O
25,000	O
50,000	O
100,000	O
150,000	O
200,000	O
250,000	O
300,000	O
400,000	O
500,000	O
600,000	O
700,000	O
800,000	O
1,000,000	O
1,200,000	O
1,400,000	O
1,700,000	O
2,000,000	O

Q2. [All 4 variants] How confident are you that the respondent understood the question in this section?

Did not understand the question at all	1	2	3	4	5	6	7	8	9	10	Fully understood the question
